# Quality of maternal and newborn healthcare services in two public hospitals of Bangladesh: identifying gaps and provisions for improvement

**DOI:** 10.1186/s12884-019-2656-1

**Published:** 2019-12-10

**Authors:** Taposh Kumar Biswas, Hasnat Sujon, M. Hafizur Rahman, Henry B. Perry, Mahbub Elahi Chowdhury

**Affiliations:** 1 International Centre for Diarrhoeal Disease Research, Bangladesh (icddr,b), 68 Shaheed Tajuddin Ahmed Sarani, Mohakhali, Dhaka, 1212 Bangladesh; 2 Johns Hopkins University Bloomberg School of Public Health, Baltimore, MD USA

**Keywords:** Bangladesh, Quality of care, Standards-based management and recognition (SBM-R), Maternal and newborn health, Public health facilities

## Abstract

**Background:**

Healthcare service delivery systems need to ensure standard quality of care (QoC) for achieving expected health outcomes. Although Bangladesh has a good healthcare service delivery system, there are major concerns about the quality of maternal and newborn health (MNH) care services, which is imperative for achievements in health. The study aimed to measure the QoC for different MNH services in two selected public health facilities of Bangladesh. This study also documented the specific areas of each care which needs intervention.

**Methods:**

The study was conducted in two district-level public health facilities—a district hospital (DH) and a mother and child welfare centre (MCWC). A total of 228 cases of MNH services were observed by using contextualized checklist ‘Standards-based Management and Recognition (S-BMR)’ for 8 selected MNH care services. For scoring, performed activities were calculated as percentages of the total recommended activities and categorized as high (> 80%), moderate (50 to 80%), and low (< 50%).

**Results:**

Overall QoC scores were moderate for each DH (54.8%), and MCWC (56.1%). In DH, the QoC score was high for blood transfusion (80.3%); moderate for maternal complications management (77.0%), caesarean section (CS) (65.6%), infection prevention (64.3%), sick newborn care (54.1%), and normal vaginal delivery (NVD) (52.6%); and low for antenatal care (ANC) (25.6%) and postnatal care (PNC) (19.0%). In MCWC, the QoC scores were high for infection prevention (83.0%); moderate for CS (76.5%) and NVD (59.8%); and low for ANC (36.9%) and PNC (24.5%).

**Conclusions:**

In the study facilities, the QoC for MNH services is found to be unsatisfactory, particularly for ANC and PNC. Urgent initiative needs to be taken by introducing contextualized quality monitoring tools at health facilities, along with training of the care providers and introducing a quality monitoring system.

## Background

Quality of care (QoC) is a central concern in health systems to improve the health status of population sustainably [1]. The World Health Organization defined quality of maternal and newborn health (MNH) care as “the degree to which maternal and newborn health services (for individuals and population) increase the likelihood of timely, appropriate care for the purpose of achieving desired outcomes that are both consistent with current professional knowledge and take into account the preferences and aspirations of individual women and their family” [2]. There is a complex relationship between quality of healthcare and expected health outcomes. However, the evidence clearly indicates that only increasing the number of facilities will not be sufficient to reduce maternal and neonatal mortality and morbidity unless QoC is maintained [3–7].

In Bangladesh, in each district, there is a district hospital (DH) and a mother and child welfare centre (MCWC). The country has a bifurcated health service delivery system where DHs belong to the Directorate General of Health Services and the MCWCs are under the Directorate General of Family Planning. Though both the directorates are under the Ministry of Health and Family Welfare, the human resources structure and management systems are different in DHs and MCWCs. Nevertheless, most of the DHs have more or less the same human resources, infrastructures, drugs, equipment and service provisions. Likewise, the district level MCWCs are similar in terms of health system readiness and services. Both types of facilities are referral hospitals and usually receive patients from sub-district and below levels of the health system [8]. However, the patients with obstetric and newborn complications are mostly referred to the DHs due to relatively better readiness to manage these complications compared to the MCWCs.

A number of studies have assessed the overall quality of MNH services in Bangladesh. Two studies reported on dissatisfaction of both clients and care providers about the existing QoC [9, 10]. Another study examined the use of different components of obstetric care services covering the use of partograph, active management of the third stage of labor, management of eclampsia, blood transfusion (BT) service, etc. [11]. However, none of the above studies critically examined the different steps of a particular service provision, which needs to be documented for a clear understanding of the specific gaps and taking necessary actions for improvement of the QoC. One recent study documented the quality of detailed process of normal vaginal delivery (NVD) [12], and another study assessed the quality of antenatal care (ANC) in primary-level health facilities [13]. However, those studies did not assess other MNH services needed during the intranatal and postnatal period.

There are several approaches for measuring the process of different MNH services systematically [14]. The Standards-based Management and Recognition (SBM-R) tool, developed by Jhpiego (Baltimore, MD, USA), has been used in several low-resource African, Asian and American countries to assess the quality of MNH services [15]. In the current study, we used this tool with an aim to quantify the quality of different MNH services systematically in two selected public health facilities of Bangladesh. The QoC of MNH services assessed included antenatal, intranatal, and postnatal care as well as management of complications and prevention of infection. This study also attempted to identify the QoC for different components of each MNH care to develop need-based interventions for ensuring optimum quality of MNH services at health facilities.

## Methods

This was a cross-sectional observational study conducted from February 2014 to May 2015 as part of an operations research to improve delivery and emergency obstetric and newborn care (EmONC) services in public facilities by health systems strengthening in Bangladesh.

### Study settings

The study facilities included a DH and a MCWC in one district with high maternal mortality in Bangladesh. Both the study facilities were secondary-level referral hospitals and designated to provide emergency obstetric and newborn care services. In the DH, there was an obstetrician and three medical doctors for providing MNH care. One anesthetist was appointed to provide anesthesia services during surgical intervention. Pediatrics services were provided by one specialist provider in both indoor and outdoor departments. Due to having no special care baby unit, sick newborn care was provided in the general paediatrics ward along with all admitted neonates and children. On the other hand, the MCWC had only one trained provider for each of obstetric and anesthesia services. In both the facilities, surgical care was provided by the obstetrician or trained medical doctors but NVDs along with ANC and PNC were mostly conducted by the nurses at DH and family welfare visitors (FWVs) in MCWC. There was no blood bank in either facility but blood collection and cross-matching services were available in DH. So, blood transfusion services were available when donor was found. The DH usually refer patients to the tertiary level health facilities i.e. medical college hospitals, and specialized hospitals. The MCWC also refer patients to the DH along with the tertiary hospitals.

### Data-collection process

The SBM-R tools were used to assess the quality of different MNH care over a period of 30 days. At the study facilities, data collection was done during the morning and evening shifts as most of the services were provided during that periods. The SBM-R tools are multifaceted checklists used to measure the quality of MNH care services, which consists of detailed systemic performance standards for assessment of clinical and support systems providing opportunity to identify the gaps [11, 12]. We contextualized the SBM-R tools according to the service provision and availability of the equipment, supplies and logistics in the study facilities. In each facility, data were collected by a team consisting of one medical doctor and one paramedic. All the members of the data-collection team received training from the study investigators on both technical and ethical aspects of the study.

The study measured the quality of 8 MNH services that included: ANC, postnatal care (PNC), normal vaginal delivery (NVD), caesarean section (CS) delivery, management of maternal complications, sick newborn care, blood transfusion (BT) service, and prevention of infection using contextualized tools (Additional files 1, 2, 3, 4, 5, 6, 7 and 8). To measure the QoC, we observed the performance of service providers to complete the activities recommended for different components of each service [2]. The number of cases observed varied from 5 to 36 for different MNH services in both the study facilities (Table 1).

**Table 1:** Number of cases observed in DH and MCWC by number of components and activities of various MNH service

Maternal and newborn services	Number of cases observed		Number of components per service	Number of maximum activities per service
	DH	MCWC		
Antenatal care	36	36	8	81
Postnatal care	20	18	11	103
Normal vaginal delivery	20	20	16	253
Caesarean section	15	14	15	152
Management of maternal complications	15	–	11	54-71^a^
Blood transfusion	10	–	4	24
Sick newborn care	14	–	5	14-20^b^
Prevention of infection	5	5	19	156

*DH* District hospital, *MCWC* Mother and child welfare centre;

^a^Number of activities varied as per type of maternal complications managed, which were incomplete abortion, pre-eclampsia/eclampsia and postpartum hemorrhage;

^b^Number of activities varied as per type of neonatal condition managed, which were preterm/low-birth-weight neonates, neonatal sepsis, and neonatal jaundice

For each MNH service, all available cases during the 30-day observation period were selected sequentially. The selection of cases depended on the patient-flow in those facilities during the observation period. In MCWC, management of maternal complications, sick newborn care, and BT services could not be observed due to unavailability of cases during the study period (Table 1).

### Data analysis

Quality measures were transformed into numerical scores. All activities were equally weighted and given a score of 1 for done, 0 for not done, and null for not applicable. QoC scores were computed as percentages of the total recommended activities that were completed. Scoring was done separately for each component, and an overall QoC score was computed for each MNH care. Then, averages of the scores of all the observed MNH services were computed to get an overall score for each facility. For interpretation, we categorized > 80% QoC scores as high, 50 to 80% as moderate, and < 50% as low.

### Ethical statement

Ethical approval of the study was obtained from the Ethics Review Committee of International Centre for Diarrhoeal Disease Research, Bangladesh (icddr,b). Informed written consents were obtained from both service providers and clients of the respective MNH services before starting observation.

## Results

**Fig. 1:**
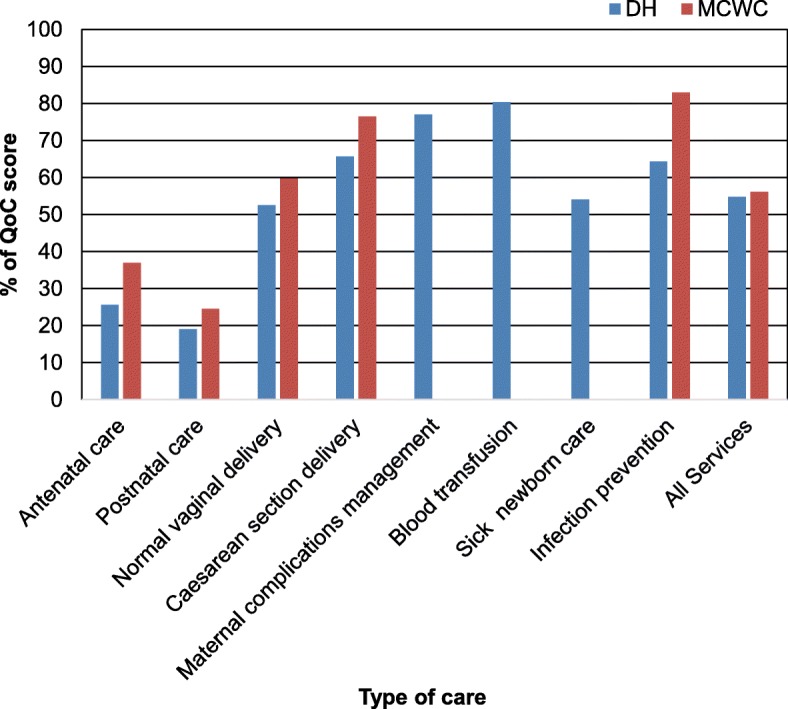
Level of QoC scores for different maternal and newborn care services by type of study facility. QoC = Quality of care, DH = District hospital, MCWC = Mother and child welfare centre

Overall QoC for MNH services were moderate in both DH and MCWC by yielding scores of 55 and 56% respectively. Though the QoC scores for CS were also moderate in both DH (65.6%) and MCWC (76.5%), the corresponding scores for ANC (DH = 25.6%, MCWC = 36.9%) and PNC (DH = 19.0%, MCWC = 24.5%) were unacceptably low. In MCWC, the QoC scores for each of the available services were relatively higher than the respective scores in DH. Although the QoC score of BT service in DH was high (80.3%), those for management of maternal complications (77.0%) and sick newborn (54.1%) care were moderate (Fig. 1).

Analysis of each of the components of specific MNH services revealed a wide variation in QoC scores in both the study facilities. In the case of ANC services, out of 8 components/activities, only 2 (‘cordial and respectful receiving’ and ‘obstetrical history-taking’) had moderate QoC scores, and the rest 6 components had low QoC scores. For ‘birth planning’ which includes planning for delivery in advance such as keeping alert skilled birth attendant, arranging emergency transport, saving fund for emergency needs etc., the QoC score was alarmingly low in both the facilities (DH = 1.5%, MCWC = 4.5%). For PNC service, the QoC scores for 10 out of 11 components were low in both the study facilities. For half of the components of PNC services (5 in DH and 6 in MCWC), the QoC scores were below 20%. In DH, the service providers failed to obtain any score in ‘management of neonates and showing breastfeeding position’ and ‘advice on baby’s danger signs’ components of PNC services (Table 2).

**Table 2:** Mean percentages of QoC scores for different components of antenatal care and postnatal care services by type of study facilities

Components of antenatal and postnatal care services	% of QoC score by type of facility			
	DH		MCWC	
	Number of activities observed^a^	Mean % of activities performed	Number of activities observed^a^	Mean % of activities performed
Antenatal care				
Rapid initial evaluation	6–7	15.5	6–7	14.8
Cordial and respectful receiving	4–5	57.1	3–5	59.4
Obstetrical history-taking	5–9	57.1	6–9	60.8
General medical history-taking	8	30.2	8	17.7
Physical and obstetric examination	10–23	15.3	6–23	57
Individualized care, based on findings and protocols	8–11	32.7	7–11	32.1
Birth planning	13	1.5	13	4.5
Care planning	5	36.1	5	48.9
Postnatal care				
Rapid initial assessment	12–13	35.3	12–13	29.0
Cordial and respectful receiving	1–2	40.0	1–2	15.6
Verification of existing clinical records or opening of new one by appropriate history-taking	4	41.3	4	19.4
Routine physical examination	14	13.6	13–14	18.3
Individualized care, based on findings and protocols	19–25	28.1	20–25	37.4
Advice on danger signs of postpartum period	9	6.1	9	13.6
Referral of mother if needed	4	50.0	b	–
Assessment of the condition of newborn	1–18	25.9	15–18	14.2
Management of neonates and showing breastfeeding position	3	0.0	3	5.9
Counseling on care of baby	9	7.2	9	34.0
Advice on baby’s danger signs	6–7	0.0	6–7	22.0

*QoC* Quality of care, *DH* District hospital, *MCWC* Mother and child welfare centre

^a^Number of applicable activities varied from case to case

^b^No case was available for this component during the study period

The QoC scores for different components of NVD were relatively poorer compared to CS. For NVD in both the study facilities, out of 16 components, only 2 (‘assist the woman to have a safe and clean birth’ and ‘neonatal resuscitation if needed’) obtained over 80% QoC scores. For 3 components of NVD (‘rapid initial assessment’, ‘use of partograph and adjustments to the birth plan’, and ‘monitoring of newborn in immediate postpartum period’), the QoC scores were < 50% in both the study facilities. For CS, out of 15 components, 6 (‘provide anesthesia’, ‘opening of abdominal layer’, ‘opening of the uterus’, ‘delivery of the baby’, ‘closure of the uterus’, and ‘closure of the abdominal wall’) achieved high QoC scores (> 80%) in both the facilities. Of the rest, 3 components (‘ensure fitness for surgery through physical and laboratory examinations’, ‘preparation of operation theater and readiness of providers for surgery’ and ‘preparation of the patients for surgery’) were high in MCWC and moderate in DH. Whereas, 4 components (‘informing the clients regarding indication, risks and benefit of CS’, ‘delivery of the placenta and exploration of the peritoneal cavity’ and ‘completion of all tasks of post procedure’, postoperative monitoring and ensure postnatal care’) were moderate in both the facilities. The QoC scores for ‘monitoring of newborn in immediate postpartum period’ was low in both the study facilities (Table 3).

**Table 3:** Mean percentages of QoC scores for different components of normal vaginal delivery and caesarean section delivery by type of study facility

Components of normal vaginal and caesarean delivery care services	% of QoC score by type of facility			
	DH		MCWC	
	Number of activities observed^a^	Mean % of activities performed	Number of activities observed^a^	Mean % of activities performed
Normal vaginal delivery				
Rapid initial assessment	9	45.6	9	46.7
Explanation of services to be provided	7	67.9	7	73.6
Review and filling-up the clinical history	16–24	55.3	13–24	57.9
Physical examinations between contractions	12–14	40.1	13–14	55.7
Obstetric examination between contractions	5	58.0	5	72.0
Vaginal examination	12–13	62.8	13	74.6
Use of partograph and adjustments to the birth plan	13–14	9.7	13–14	11.0
Preparation for assisting birth	11	42.7	11	74.5
Assist the woman to have a safe and clean birth	16–22	80.8	16–22	85.8
Initial assessment of the newborn and providing immediate newborn care	8–11	78.6	8–11	78.4
Active management of the third stage of labor	16–17	63.0	16–17	71.5
Immediate postpartum care	6–12	68.7	6–12	89.7
Disposal of used instruments and medical waste	10	43.5	10	76.5
Monitoring of newborn in immediate postpartum period	10–37	32.8	33–37	33.6
Close monitoring of the woman for at least two hours after the childbirth	37–39	49.5	38–39	54.8
Neonatal resuscitation if needed	1–13	86.7	4–13	100.0
Caesarean section (CS) delivery				
Informing the clients regarding indication, risks and benefit of CS	13–14	51.7	13–14	65.7
Ensure fitness for surgery through physical and laboratory examinations	5	52.0	4–5	81.4
Preparation of operation theater and readiness of providers for surgery	11	70.9	11	83.8
Preparation of the patients for surgery	7	62.9	7	88.8
Provide anesthesia	7–8	84.0	8	95.5
Opening of the abdominal layer	8	84.2	8	100.0
Opening of the uterus	7–9	90.0	8–9	92.0
Delivery of the baby	7	80.0	7	89.8
Delivery of the placenta and exploration of the peritoneal cavity	4–7	58.1	5–7	68.0
Closure of the uterus	7–9	88.5	8–9	96.0
Closure of the abdominal wall	6	98.9	6	100.0
Completion of all tasks of post procedure	8	55.8	8	77.7
Postoperative monitoring and ensure postnatal care	8–16	71.2	10–16	79.2
Monitoring of newborn in immediate postpartum period	37	34.1	36–37	33.8
Resuscitation of newborn if needed	b	–	2	50.0

*QoC* Quality of care, *DH* District hospital, *MCWC* Mother and child welfare centre

^a^Number of applicable activities varied from case to case

^b^No case was available for this component during the study period

Regarding management of maternal complications, 5 out of 11 components obtained high QoC scores. The QoC scores were moderate for the 5 other components (‘evaluation of patient’s response and next step’, ‘management of incomplete abortion’, ‘management of severe pre-eclampsia and/or eclampsia’, ‘general management of PPH’, and ‘follow-up of the PPH patient’) but low in ‘cause-specific management of PPH’ (Table 4).

**Table 4:** Mean percentages of QoC scores for different components of maternal and newborn complications management and blood transfusion services in DH

Components of maternal and newborn complications management and blood transfusion services	% of QoC score	
	Number of activities observed^a^	Mean % of activities performed
Management of maternal complications		
Availability of drugs, equipment, and supplies	4	89.5
Management of hypovolemic shock	4–14	81.9
Evaluation of patient’s response and next step	6–11	67.7
Diagnosis of incomplete abortion	6–7	90.5
Management of incomplete abortion	5–8	68.8
Diagnosis of pre-eclampsia/eclampsia	3–4	91.7
Management of severe pre-eclampsia and/or eclampsia	11–16	76.0
Diagnosis of PPH	1	100.0
General management of PPH	14–17	62.3
Cause-specific management of PPH	1–12	49.0
Follow-up of the PPH patient	6–8	59.9
Sick newborn care		
Receiving patient, history-taking, and explanation of the condition	5	70.0
Appropriate diagnosis and management of a preterm/low-birth-weight neonate	12–15	31.7
Diagnosis of neonatal sepsis and appropriate referral if required	8–9	53.0
Diagnosis of neonatal jaundice and appropriate referral if required	6–9	51.9
Blood transfusion services		
Cordial receiving of the patient and response to any query	4	55.0
Assessment of the donor for fitness of blood donation	4	75.0
Blood-collection procedure	6	91.7
Appropriate blood transfusion procedure	9–10	86.8

*QoC* Quality of care, *DH* District hospital, *MCWC* Mother and child welfare centre, *PPH* Postpartum hemorrhage

^a^Number of applicable activities varied from case to case

The QoC scores for the majority (3 out of 4) of the components of sick newborn care were moderate. For ‘appropriate diagnosis of a preterm/low-birth-weight neonate’, only 31.7% score was achieved. For half of the components of BT service (‘cordial receiving of the patient and response to any query’ and ‘assessment of the donor for fitness of blood donation’), the QoC scores were either moderate or low (Table 4).

As shown in Table 5, the QoC scores for most of the components of the prevention of infection were relatively high in the MCWC compared to those in DH. Again, QoC scores for prevention of infection in the labor rooms were lower than the corresponding figures in operation theaters in the respective facilities. In DH, the QoC scores were < 50% for 4 components (‘process to clean rooms, wards and clinical areas’, ‘decontamination of equipment for re-use or storage in labor room’, ‘use of antiseptics in labor room’, and ‘collection of soiled linen’).

**Table 5:** Mean percentages of QoC scores for different components of infection prevention services by type of facility

Components of infection prevention	% of QoC score by type of facility			
	DH		MCWC	
	Number of activities observed^a^	Mean % of activities performed	Number of activities observed^a^	Mean % of activities performed
Cleanliness of facility and availability of clean running water	12–14	52.6	13–14	91.2
Process to clean rooms, wards, and clinical areas	23	41.7	23	70.4
Decontamination of equipment for re-use or storage				
Labor room	5	48.0	5	88.0
Operation theater	5	80.0	5	100.0
Use of antiseptics				
Labor room	7	40.0	7	62.9
Operation theater	7	60.0	7	82.9
Instrument cleaning area				
Labor room	9	77.8	9	80.0
Operation theater	9	86.7	9	91.1
Decontamination of instruments				
Labor room	5	56.0	5	84.0
Operation theater	5	72.0	5	80.0
Process of cleaning instruments				
Labor room	7	77.1	7	94.3
Operation theater	7	80.0	7	94.3
Area for wrapping and packing instruments	3	60.0	3	66.7
Process of packaging of instruments and other items for sterilization	4	80.0	4	95.0
Sterilization process	17–18	71.8	16–18	91.6
High-level disinfection process	4	90.0	4	100.0
Availability of antiseptics, disinfectants, and other supplies	14	61.4	14	78.6
Collection of soiled linen	3	26.7	3	40.0
Following of general biosafety and infection prevention practices in the laboratory	7	97.1	b	–

*QoC* Quality of care, *DH* District hospital, *MCWC* Mother and child welfare centre

^a^Number of applicable activities varied from case to case

^b^As no laboratory service was available in MCWC

## Discussion

Our study demonstrated that there were major deficiencies in the quality of MNH services in both the study facilities. Overall, the service providers in these facilities failed to follow about half of the standard activities of MNH care. In the case of maternal care, the quality was low for ANC and PNC; moderate for NVD, CS and complication management in both the facilities. In the DH, the quality of blood transfusion was high but that of infection prevention and sick newborn care were moderate and low respectively.

The QoC scores for both ANC and PNC were unacceptably low in each DH and MCWC. For both of these services, the QoC scores for ‘rapid initial evaluation’, ‘cordial and respectful receiving’, ‘history-taking’, ‘physical examination’ and ‘individualized care’ were far from satisfactory. Quality was surprisingly low for ‘birth planning’ component in ANC and for ‘neonatal management’ component in PNC. Providers in both the facilities rarely advised on ‘proper breastfeeding technique’ and informed about ‘maternal and neonatal danger signs’. A separate study in Bangladesh, using standard operation procedures of the Directorate General of Health Services [13], documented relatively high quality of ANC service compared to our study. However, that study was conducted in primary health facilities, and the variations in findings might be due to difference in the context.

Lack of refresher training and inadequate human resources might have contributed to the poor quality of care in secondary facilities [10]. Since the quality of ANC influences pregnancy outcomes [16] and a high burden of maternal and neonatal mortality exists during the postnatal period [17], standard QoC needs to be maintained. In addition to increasing human resource, special orientation programmes and refresher training for the service providers with emphasis on the low scoring areas might be useful for improving the quality of ANC and PNC.

Although the service providers in both the facilities of our study could perform more than half and three-fourths of the total standard activities while providing NVD and CS delivery services respectively, there is still considerable room for further improvement of the quality of these services. Providers in these facilities performed poorly in providing newborn care immediately after NVD and CS delivery. For NVD, the quality fell due to poor performance in ‘rapid initial assessment’ and ‘physical examination’. More than one-third of the activities in ‘active management of the third stage of labor’ that needed to be addressed properly for prevention of PPH, was not performed [18]. The QoC scores for using partograph in both the facilities were extremely poor. Similar findings were also documented in a previous study [12]. The care providers might not be in a position to use the labor-monitoring tool due to lack of training and high patient-load [19]. Our study found that clients were inadequately counseled on indications, risks, and benefits of CS. Another study revealed that there was skepticism among clients about the service providers’ justification of CS in Bangladesh [20]. To improve the situation, adequate training should be provided to human resources with emphasis on clinical assessment and monitoring. Advocacy program needs to be endorsed to motivate the service providers for using labour monitoring tool like partograph in order to observe progress in labour and foetal condition and make decision for appropriate intervention.

Standards of quality must be maintained while managing maternal and newborn complications to reduce the risk of serious complications and avert death. We found that the service providers completed most of the activities in general management of maternal complications and diagnosis of specific causes. However, the quality of management of specific conditions, such as pre-eclampsia/eclampsia and incomplete abortion was deficient. Poor quality of PPH management is a serious concern as it is the single-most important cause of maternal mortality [21]. Most prevalent causes of neonatal deaths included prematurity, birth complications (birth asphyxia and trauma) and sepsis [22]. Our findings on poor QoC in identifying specific newborn complications and lacking in providing appropriate care are similar to those from another study in Bangladesh [23]. We also documented substantially low performance in appropriate diagnosis of preterm and low-birth-weight neonates. Quality of diagnosis and referral for neonatal sepsis and jaundice were low mainly due to care providers’ poor practice to recognize the signs/symptoms of these complications. The current low qualities of sick newborn care service at these district-level facilities in Bangladesh need to be improved through special training program of the service providers. While assessing BT service, we found that standards in receiving the clients and assessing the blood donor for fitness were not maintained properly and that may enhance the risk of getting infected by intravenously transmitted diseases among the patients. Intermittent advocacy program and refresher training of the service providers may help overcome these inadequacies.

Although overall QoC scores for prevention of infection was better in the MCWC, component-wise analysis revealed that the quality of the ‘use of antiseptics in labor room’; ‘availability of the antiseptics, disinfectants, and other supplies’; and ‘collection of soiled linen’ was inadequate in both the study facilities. These shortcomings, along with the low score for sterilization processes in DH, increase the risk of hospital-acquired infection of mothers and newborns. The main cause of hospital-acquired infection is the substandard practice for prevention of infection and simple strategies, such as hand washing which can reduce this burden significantly [24]. As prevention of infection is a broad issue; the support staff of the hospitals, along with the care providers, should be appropriately trained to ensure proper sterility. Behavior change education, monitoring and supportive supervision of the staff may improve the situation [25].

One limitation of our study is that we did not correlate the QoC with availability of human resources, functioning equipment, logistics and supplies. However, the poor quality of primary MNH care in the study facilities is likely to be due to inadequate human resource and high patient load that had been documented in our another paper [26] developed from the same study. This also has been confirmed by the service providers while sharing the study findings with them. In addition to training of the existing manpower, they suggested to ensure availability of adequate human resources to practice the QoC protocols for various MNH care services. Another limitation of the study is not considering the delay in providing services at health facilities. Further qualitative studies are needed to explore the impact of delay in providing services on quality of MNH services. In this study, we observed the patients for QoC only during the morning and shifts. Not observing services at the night shift does not likely to affect the findings as the services are scanty is public facilities during that period. Only one month observation time period was another limitation in capturing adequate number of infrequent services (such as complication management, infection prevention and BT). This short observation period also did not allow us to mitigate the seasonal dips. Above all, we did not apply weights for different activities of various MNH services, as there is no standard available in the literature.

Substandard QoC in the public health sector in Bangladesh might have contributed to the recent lack of progress in health indicators [4]. The Government has developed national healthcare standards on QoC [27] but these are yet to be implemented and lack many important technical details. There is a need to develop a contextualized facility-specific quality monitoring tool of MNH service by reviewing SBM-R and other existing methods through expert consultation. Self-implementation of the newly-developed quality assessment tool can be a way of improving the QoC, and, for that purpose, training activities and motivation programs for service providers should be undertaken.

## Conclusions

In Bangladesh, QoC for MNH services in the DH and MCWC is much below the acceptable level. The QoC is alarmingly low for the ANC and PNC services. To improve the situation, there is an urgent call for developing facility-specific contextualized tools and implement those through appropriate training and supportive supervision for maintaining minimum required quality of MNH care. An external quality-monitoring team should be vigilant to ensure accountability of the health facilities in providing quality services.

## Supplementary information

**Additional file 1.** Checklist _Normal Vaginal Delivery.doc (Nomal delivery checklist).

**Additional file 2.** Checklist_ANC.docx (ANC Checklist).

**Additional file 3.** Checklist_Blood Transfustion.docx (Blood transfussion checklist).

**Additional file 4.** Checklist_Caesarean Section Delivery.doc (Cesarean delivery checklist).

**Additional file 5.** Checklist_Infection Orevention.doc (Infection prevention checklist).

**Additional file 6.** Checklist_Management of Maternal Complications.docx (Maternal complications management checklist).

**Additional file 7.** Checklist_PNC.doc (PNC checklist).

**Additional file 8.** Checklist_Sick Newborn Care.docx (Sick newborn care checklist).

## Data Availability

The detail dataset is available with MEC, the Principal Investigator of the main study. A copy of the original data is also stored in the data archive of icddr,b. These data are not publicly available. However, non-identifiable data can be accessible upon request subject to approval of the Research Administration Department of icddr,b.

## Abbreviations

ANC: Antenatal care
BT: Blood transfusion
CS: Caesarean section
DH: District hospital
icddr,b: International centre for diarrhoeal disease research, Bangladesh
MCWC: Mother and child welfare centre
MNH: Maternal and newborn healthcare
NVD: Normal vaginal delivery
PNC: Postnatal care
QoC: Quality of care
SBM-R: Standards-based management and recognition
