# Supplementary material for: Quality of maternal and newborn healthcare services in two public hospitals of Bangladesh: identifying gaps and provisions for improvement
Source: BMC Pregnancy Childbirth. 2019 Dec 10;19:488. doi: 10.1186/s12884-019-2656-1 (PMC6905111; doi:10.1186/s12884-019-2656-1)
Supplement: Supplementary file 1 — Additional file 1. Checklist _Normal Vaginal Delivery.doc (Nomal delivery checklist). [file 12884_2019_2656_MOESM1_ESM.doc]

**Appendix V: Checklists to observe quality of care of MNH Cases attending the Health Facility**

**International Centre for Diarrhoeal Disease Research, Bangladesh (icddr,b)**

**AREA 3: NORMAL LABOR, CHILDBIRTH AND IMMEDIATE NEWBORN CARE**

**Facility Name: _____________________________________ Facility Type: ______________________________**

**District: _________________________________ Upazilla: _____________________**

**UFI of the facility:** |___|___|___|___|___|___|___|___|

**Place of observation: ____________________________________**

**Code list:** 01= OPD/EPI room, 02=Ward/Cabin, 03=ANC room, 04=Labor/Delivery room, 05=OT,

06=Nurse/ SACMO/CHCP Room, 07= Others (specify_______________________________)

**Assessment Type:** (BASELINE 🞎/PERIODIC¨)

**Phase of Data collection:** Phase I 🞎/Phase II 🞎/Phase III 🞎

**Name of the Observer** ___________________________________

**Case no:** |___|___| **Patient no:** |___|___|___|___|

**Date:** ___/___/2014  **Observation Start Time: |___||___|:|___||___|**

**Operational definition:**

**Done:** Performs the step or task according to the standard procedure or guidelines.

**Not done**: Unable to perform the step or task according to the standard procedure or guidelines.

**Not applicable**: Step or task not applicable for that particular patient during evaluation by observer.

| **PERFORMANCE STANDARDS** | **Sl. No.** | **VERIFICATION CRITERIA** | | | **Observation**  **[Done=1,**  **Not done=0,**  **Not applicable=9** | | **COMMENTS** |
| --- | --- | --- | --- | --- | --- | --- | --- |
| **RAPID INITIAL ASSESSMENT OF PREGNANT WOMEN IN LABOR BEFORE ADMISSION** | | | | | | | |
| 1. The facility has a system to perform a rapid initial assessment of the pregnant women in labor to identify complications and prioritize admissions/makes decision accordingly. (Observe in the registration/admission and/or in the examination room, or in the emergency room, during a period of time that allows you to see more than one woman in labor) | 1.1 | When assessing individually each woman the provider:  Determines if birth is imminent (desire to bear down, perspiration, anxiety) | | |  | |  |
|  | 1.2 Asks the woman whether she has or has had: | | | | | | |
|  | 1.2.a | Vaginal bleeding | | |  | |  |
|  | 1.2.b | Rupture of membranes | | |  | |  |
|  | 1.2.c | Convulsions | | |  | |  |
|  | 1.2.d | Severe headache and blurred vision | | |  | |  |
|  | 1.2.e | Severe abdominal pain | | |  | |  |
|  | 1.2.f | Respiratory difficulty | | |  | |  |
|  | 1.2.g | Fever | | |  | |  |
|  | 1.3 | Records the information on woman’s clinical history and refers women after providing immediate treatment if required | | |  | |  |
|  | **Achieved: Yes/No (Circle the answer)** | | | | | |  |
| 2. The provider treats the pregnant woman in labor in a cordial manner. (Observe one woman in labor and determine whether the provider (in the labor and delivery rooms): | 2.1 | Greets the woman and her husband or companion in a cordial manner and introduces her/himself | | |  | |  |
|  | 2.2 | Ensures that she/he speaks in easy to understand language with the client | | |  | |  |
|  | 2.3 | Tell the mother and her support person what is going to be done and encourage them to ask questions) | | |  | |  |
|  | 2.4 | Listen to what the mother and her support person have to say | | |  | |  |
|  | 2.5 | Responds to questions and concerns | | |  | |  |
|  | 2.6 | Provide emotional support and reassurance | | |  | |  |
|  | 2.7 | Responds to her immediate needs (thirst, hunger, cold/hot, need to urinate, etc.) | | |  | |  |
|  | **Achieved: Yes/No (Circle the answer)** | | | | | |  |
| 3. The provider properly reviews and fills out the clinical history of the woman in labor. (Observe one woman in labor and determine whether the provider (in the labor room) | 3.1 | Asks and records on the woman’s clinical history the following information in a confidential and private environment. | | |  | |  |
|  | 3.2 | Age of mother | | |  | |  |
|  | 3.3 Previous obstetric history | | | | | | |
|  | 3.3.a | no.of pregnancies | | |  | |  |
|  | 3.3.b | no.of abortions | | |  | |  |
|  | 3.3.c | no. of normal deliveries | | |  | |  |
|  | 3.3.d | no. of caesarean sections | | |  | |  |
|  | 3.3.e | no. of children born alive | | |  | |  |
|  | 3.3.f | no. of still birth | | |  | |  |
|  | 3.3.g | Any maternal complication in any previous pregnancy | | |  | |  |
|  | 3.3.h | date and outcome of last pregnancy | | |  | |  |
|  | 3.4 Other general medical problems (for diabetes, hypertension, Asthma etc.) | | | | | | |
|  | 3.4.a | Any medication | | |  | |  |
|  | 3.4.b | Use of alternative medications or herbs | | |  | |  |
|  | 3.4.c | Receiving treatment for Tuberculosis. | | |  | |  |
|  | 3.5 Gestational age – ask or calculate | | | | | | |
|  | 3.5.a | last menstrual period (LMP) | | |  | |  |
|  | 3.5.b | Expected Date of Delivery (EDD) | | |  | |  |
|  | 3.6 | Avoids asking questions during contractions | | |  | |  |
|  | 3.7 | Asks the woman about her labor/the painful regular contractions began | | |  | |  |
|  | 3.8 | Frequency and strength of the contractions | | |  | |  |
|  | 3.9 If her “bag of waters” broke/membrane ruptured? | | | | | | |
|  | 3.9.a | When the water broke?? | | |  | |  |
|  | 3.9.b | What color was the liquor? | | |  | |  |
|  | 3.9.c | What smell the liquor had? | | |  | |  |
|  | 3.9.d | Presence of vaginal bleeding | | |  | |  |
|  | 3.10 | Whether she feels the baby’s movements | | |  | |  |
|  | 3.11 | Records the information on clinical history form | | |  | |  |
|  | **Achieved: Yes/No (Circle the answer)** | | | | | |  |
| 4. The provider properly conducts the physical examination between contractions and if time allows. | 4.1 | Help the mother into the examination table | | |  | |  |
|  | 4.2 Ensures privacy during the entire process of provision of care: | | | | | | |
|  | 4.2.a | Ensures privacy with a screen or curtain to separate the woman from others, at least during examination | | |  | |  |
|  | 4.2.b | Ensures that the woman remains covered with her robe or clothing | | |  | |  |
|  | 4.3 | Asks the woman to urinate (tests urine for albumin and sugar) if necessary or if not done before | | |  | |  |
|  | 4.4 | Washes hands | | |  | |  |
|  | 4.5 | Explains each step of the examination to the woman using easy-to-understand language | | |  | |  |
|  | 4.6 Takes or delegates vital signs to assistant | | | | | | |
|  | 4.6.a | Temperature | | |  | |  |
|  | 4.6.b | Blood pressure (BP) | | |  | |  |
|  | 4.6.c | pulse | | |  | |  |
|  | 4.6.d | Respiratory rate | | |  | |  |
|  | 4.6.e | Oedema | | |  | |  |
|  | 4.6.f | Checks the conjunctiva and palms of hands for anemia | | |  | |  |
|  | 4.6.g | tests blood for hemoglobin if anemia suspected | | |  | |  |
|  | 4.7 | Observe the mother’s emotional response to labor | | |  | |  |
|  | **Achieved: Yes/No (Circle the answer)** | | | | | |  |
| 5. The provider properly conducts the obstetric examination between contractions if time allows. | 5.1 | Observes the shape and size of the abdomen and checks for the presence of scars | | |  | |  |
|  | 5.2 | Determines fundal height, presentation and if necessary, fetal lie | | |  | |  |
|  | 5.3 | Evaluates uterine contractions (frequency and duration over a 10-minute period) | | |  | |  |
|  | 5.4 | Auscultates fetal heart rate (FHR) | | |  | |  |
|  | 5.5 | Records the results of the obstetric examination on the clinical history | | |  | |  |
|  | **Achieved: Yes/No (Circle the answer)** | | | | | |  |
| 6. The provider properly conducts a vaginal examination. | 6.1 | Put on a clean plastic or rubber apron | | |  | |  |
|  | 6.2 | Performs hand hygiene and sterile/HLD gloves on both hands | | |  | |  |
|  | 6.3 | Examines the vulva (ulcers, blood, liquid, secretion, presenting part) | | |  | |  |
|  | 6.4 | Cleanses the perineum with nonalcoholic antiseptic solution using the hand that will not be used to perform the examination | | |  | |  |
|  | 6.5 Performs per vaginal examination following standard technique | | | | | | |
|  | 6.5.a | Gently insert two lubricated fingers of the examining hand into the vagina | | |  | |  |
|  | 6.5.b | Note the length, consistency and dilatation of the cervix | | |  | |  |
|  | 6.5.c | Note the membranes are intact, and if they have ruptured, ensure the cord has not prolapsed | | |  | |  |
|  | 6.5.d | Measure the level of the presenting part in centimeters above or below the mother’s ischial spines | | |  | |  |
|  | 6.5.e | Identify the presentation and determine the position by feeling the features of the presenting part | | |  | |  |
|  | 6.5.f | Immerses both gloved hands in a 0.5% chlorine solution, and removes gloves by turning inside out, | | |  | |  |
|  | 6.6 | If gloves are disposable, places them in a waste-container with a plastic liner; OR  if they are reusable, immerses them in a 0.5% chlorine solution for at least 10 minutes before transferring them for sterilization | | |  | |  |
|  | 6.7 | Performs hand hygiene after removing gloves | | |  | |  |
|  | 6.8 | Record the results of the vaginal examination on the clinical history | | |  | |  |
|  | **Achieved: Yes/No (Circle the answer)** | | | | | |  |
| 7. The provider uses the partograph to monitor labor and make adjustments to the birth plan. (Observe whether the provider uses the partograph and.) | 7.1 | Record patient information:Name, Gravida, para, Hospital number, Date and time of admission, | | |  | |  |
|  | 7.2 | Record Time of ruptured membranes | | |  | |  |
|  | 7.3 | Records every half hour - FHR, | | |  | |  |
|  | 7.4 | Records every half hour - Uterine contractions (frequency and duration over a 10-minute period), | | |  | |  |
|  | 7.5 | Records every half hour - Maternal pulse | | |  | |  |
|  | 7.6 | Records mother’s BP every four hours | | |  | |  |
|  | 7.7 Conduct vaginal examination at every four hours or less according to evolution of labor.  At every vaginal examination - | | | | | | |
|  | 7.7.a | Records the condition of the membranes and characteristics of the amniotic fluid | | |  | |  |
|  | 7.7.b | Graphs the degree of molding of the head | | |  | |  |
|  | 7.7.c | Graphs cervical dilation | | |  | |  |
|  | 7.7.d | Graphs the descent of the head or buttocks | | |  | |  |
|  | 7.8 | Records the amount of urine output. | | |  | |  |
|  | 7.9 | Records the time of the above observations. | | |  | |  |
|  | 7.10 | Adjusts the labor plan according to the parameters encountered: | | |  | |  |
|  | 7.11 | If parameters are normal, continues to implement the plan (walk about freely, hydration, light food if desired, change positions, etc.) OR  If parameters are not normal, identifies complications, records the diagnosis and makes adjustments to the birth plan | | |  | |  |
|  | **Achieved: Yes/No (Circle the answer)** | | | | | |  |
| 8. The provider prepares to assist the birth. [Observe one woman in labor and determine whether the provider (in the labor or delivery rooms) has prepared the following equipment] | 8.1 | Birth kit comprising following -Sterile tray, Two hemostats (clamps), One scissors , One cord clamp or sterile tape or three sterile tie, two sterile/clean cloths (for baby), Sterile gauze to clean baby’s mouth and nose, One syringe with 10 IU of oxytocin, Two pairs of sterile or HLD gloves,  Ensures that the episiotomy kit is available in the labor room (for cases who require episiotomy, but not for all cases/primis) | | |  | |  |
|  | 8.2 | One plastic container with 0.5% chlorine solution for decontamination | | |  | |  |
|  | 8.3 | One plastic container with a plastic liner to dispose the placenta | | |  | |  |
|  | 8.4 | One plastic container with a plastic liner for medical waste (gauze, swab, etc.) | | |  | |  |
|  | 8.5 | One sharps container at point of use to dispose of needle and syringe | | |  | |  |
|  | 8.6 | One leak-proof container to dispose of soiled linen | | |  | |  |
|  | 8.7 | Clean plastic or rubber apron and face shields (or mask and goggles) | | |  | |  |
|  | 8.8 | Rubber shoes/sandals | | |  | |  |
|  | 8.9 | Allows the woman to give birth in the position she wants and in the same bed where she has labored (if possible) | | |  | |  |
|  | 8.10 | Allow the mother to push spontaneously | | |  | |  |
|  | 8.11 | ensures that the minimum number of individuals are present during birth (the provider attending the birth and a family member—the individual chosen by the woman) | | |  | |  |
|  | **Achieved: Yes/No (Circle the answer)** | | | | | |  |
| 9. The provider assists the woman to have a safe and clean birth. (Observe one woman during a delivery and determine whether the provider (in the labor or delivery rooms): | 9.1 | Washes hands and thoroughly with soap and water and dry them | | |  | |  |
|  | 9.2 | Puts sterile or HLD gloves on both hands | | |  | |  |
|  | 9.3 | Puts on personal protective equipment (Plastic/rubber apron, eye protection, masks and gloves) | | |  | |  |
|  | 9.4 | Cleanses the perineum with water or a nonalcoholic antiseptic solution | | |  | |  |
|  | 9.5 | Monitors, or has assistant monitor, FHR every five minutes during second stage | | |  | |  |
|  | 9.6 | Allows the woman to bear down when she feels the desire (does not force her to bear down) | | |  | |  |
|  | 9.7 | Performs an episiotomy only if necessary (breech, shoulder dystocia, forceps, vacuum, poorly healed 3rd or 4th degree tear, or fetal distress) as the perineum distends | | |  | |  |
|  | 9.8 | Administer local anesthesia. | | |  | |  |
|  | 9.9 | Wait to perform episiotomy until the perineum is thinned out and the baby’s head is visible during a contraction. | | |  | |  |
|  | 9.10 | If delivery of the head does not follow immediately, apply pressure to the episiotomy site between contractions. | | |  | |  |
|  | 9.11 | Allows the head to spontaneously crown while guarding the perineum and episiotomy (if given) | | |  | |  |
|  | 9.12 | After the emergence of the head, asks the woman to briefly refrain from bearing down (open mouth breathing) | | |  | |  |
|  | 9.13 | Assists in delivering the baby: after crowning allow the head to gradually extend and Quickly palpates to determine cord around the neck;  - if it is loose, slides it over the baby’s head;  - if it is very tight, clamps it in two places and cuts it before unravelling it from around the baby’s neck | | |  | |  |
|  | 9.14 | Assist the mother for delivering the baby - Allows spontaneous restitution and external rotation of the head without manipulation | | |  | |  |
|  | 9.15 | guides the baby’s head and chest in an upward curve until the posterior shoulder has emerged over the perineum, | | |  | |  |
|  | 9.16 | Holds the baby around the chest to aid the birth of the trunk and lift it towards the mother’s abdomen | | |  | |  |
|  | 9.17 | places it on a dry towel/cloth on the mother’s abdomen | | |  | |  |
|  | 9.18 | Cleans the baby’s mouth and nose using a sterile gauze if meconium present | | |  | |  |
|  | 9.19 | Wipe the baby’s eyes with a clean piece of cloth | | |  | |  |
|  | 9.20 | Clamps and cuts the cord using clean/sterile blade/ instrument within 1-3 minutes of the birth and cut it close to the perineum | | |  | |  |
|  | 9.21 | | Informs mother of the sex of her child (with same enthusiasm if male or female) |  | |  | |
|  | 9.22 | Passes the wrapped baby to mother for skin-to-skin contact on breast and to initiate breastfeeding | | |  | |  |
|  | **Achieved: Yes/No (Circle the answer)** | | | | | |  |
| 10. The provider properly conducts a rapid initial assessment and provides immediate newborn resuscitation if needed. (Observe one woman with her newborn in the immediate postpartum and determine whether the provider (in the labor or delivery rooms)  Note 1: This standard must be observed immediately following birth.  Note 2: It may be necessary to have two observers in the event that one provider is caring for the woman and the other for the newborn. | 10.1 | | Receives the baby either by a different provider with separate gloves or by same provider using a new set of gloves |  | |  | |
|  | 10.2 | dries the baby with a clean dry towel from head to feet, | | |  | |  |
|  | 10.3 | discards the used towel and covers the baby including the head with a clean dry towel. | | |  | |  |
|  | 10.4 | Determines whether the baby is breathing | | |  | |  |
|  | 10.5 If the baby does not begin breathing or is breathing with difficulty | | | | | | |
|  | 10.5.a | asks assistance | | |  | |  |
|  | 10.5.b | rapidly ties and cuts the cord | | |  | |  |
|  | 10.5.c | initiates resuscitation- **see newborn resuscitation goes to question # 16** | | |  | |  |
|  | 10.6 | If the baby is breathing normally, place the baby in skin-to-skin contact on the mother’s chest or abdomen | | |  | |  |
|  | 10.7 | Encourages “Baby Crawl” practice and immediate breastfeeding | | |  | |  |
|  | 10.8 | Notes the date and time of delivery | | |  | |  |
|  | 10.9 | Informs the attendant of the baby’s condition | | |  | |  |
|  | **Achieved: Yes/No (Circle the answer)** | | | | | |  |
| 11. The provider adequately performs active management of the third stage of labor. (Observe one woman during a delivery and determine whether the provider (in the labor or delivery rooms) | 11.1 | Touches the mother’s abdomen to rule out the presence of a second baby (without stimulating contractions) | | |  | |  |
|  | 11.2 | Give Inj. Oxytocin 10 units direct I/M and wait for next contraction within one minute of delivery of the baby. | | |  | |  |
|  | 11.3 | Give Inj. Oxytocin 10 units in 1 litre I/V drip @of 60 drops/min for 2 hours within one minute of delivery of the baby. | | |  | |  |
|  | 11.4 | Place a sterile receptacle (kidney basin) against the mother’s perineum | | |  | |  |
|  | 11.5 | Hold the cord along with clamp & provide controlled cord traction/sustained downward traction | | |  | |  |
|  | 11.6 | Repeats controlled cord traction while simultaneously applying counter pressure above pubis to guard uterus (Place the other hand above the level of the symphysis pubis with palmer surface facing towards the umbilicus to provide counter traction on the uterus) | | |  | |  |
|  | 11.7 | Apply steady tension by pulling the cord firmly and maintaining pressure (jerky movements and force must be avoided) | | |  | |  |
|  | 11.8 | When the placenta is visible at the vulva hold the placenta with both hands, assists in the expulsion of the placenta, by turning it over in the hands, without applying traction, “teasing out” the membranes | | |  | |  |
|  | 11.9 | Use a gentle and upward and downward movement or twisting action to deliver the membranes | | |  | |  |
|  | 11.10 | Check whether the uterus is well contracted | | |  | |  |
|  | 11.11 | Massages the uterus with one hand on a sterile cloth over the abdomen, until the uterus contracts firmly | | |  | |  |
|  | 11.12 | Check whether all of the lobules are present and fit together to see if complete (missing cotyledon) and to identify any abnormalities | | |  | |  |
|  | 11.13 | Inspect the membranes for completeness | | |  | |  |
|  | 11.14 | Note the position of insertion of the cord | | |  | |  |
|  | 11.15 | Inspect the cut end of the cord for the presence of two arteries and one vein. | | |  | |  |
|  | 11.16 | Note the insertion of the cord and examine the cut end of the cord | | |  | |  |
|  | 11.17 | Continue uterine message every 15 minutes up to two hours | | |  | |  |
|  | **Achieved: Yes/No (Circle the answer)** | | | | | |  |
| 12. The provider adequately performs immediate postpartum care (Observe women during delivery and determine) in the labor or delivery rooms immediately after delivery | 12.1 | Ask assistant to direct a strong light into the perineum | | |  | |  |
|  | 12.2 | Informs the woman what she is going to do before proceeding, then carefully examines the vagina and perineum | | |  | |  |
|  | 12.3 | Inspect the lower vagina and perineum for lacerations/tear | | |  | |  |
|  | 12.4 | Gently cleanses the vulva and perineum with clean water or a nonalcoholic antiseptic solution and dry with a clean, soft cloth | | |  | |  |
|  | 12.5 | Sutures tears/episiotomy, if necessary | | |  | |  |
|  | 12.6 | Use a continuous suture from the apex downward to repair the vaginal incision. | | |  | |  |
|  | 12.7 | At the vaginal opening, bring the cut edges together. | | |  | |  |
|  | 12.8 | Use interrupted sutures to repair the perineal muscle, working from the top of the perineal incision downward. | | |  | |  |
|  | 12.9 | Use interrupted or subcuticular sutures to bring the skin edges together. | | |  | |  |
|  | 12.10 | Covers the perineum with a clean cloth/sanitary pad | | |  | |  |
|  | 12.11 | Remove soiled bedding/linen | | |  | |  |
|  | 12.12 | Makes sure that the woman is comfortable (clean, hydrated and warmly covered) | | |  | |  |
|  | **Achieved: Yes/No (Circle the answer)** | | | | | |  |
| 13. The provider properly disposes of the used instruments and medical waste after assisting the birth. (Observe one woman in the immediate postpartum and determine whether the provider or assistant (in the labor or delivery rooms), while wearing gloves) | 13.1 | Dispose the placenta by incineration OR  Discards the placenta in a leak-proof container with a plastic liner for burial | | |  | |  |
|  | 13.2 | Puts the soiled linen in a leak-proof container | | |  | |  |
|  | 13.3 | Disposes of medical waste (gauze, swabs, other waste materials etc.) in a plastic container with a plastic liner | | |  | |  |
|  | 13.4 | Before removing gloves, place soiled linen in 0.5% chlorine solution for 10 minutes for decontamination | | |  | |  |
|  | 13.5 | Opens (un-hinges) all instruments and immerses them in a 0.5% chlorine solution for 10 minutes for decontamination | | |  | |  |
|  | 13.6 | Decontaminate or dispose of syringe and needle:  If reusing needle or syringe, fill syringe (with needle attached) with 0.5% chlorine solution and submerge in solution for 10 minutes for decontamination OR  If disposing of needle and syringe, flush needle and syringe with 0.5% chlorine solution three times, then place in a puncture-proof container | | |  | |  |
|  | 13.7 | Wipes down all surfaces with 0.5% chlorine solution | | |  | |  |
|  | 13.8 | If gloves are disposable, immerses both gloved hands in a 0.5% chlorine solution, removes gloves by turning inside out, and  If disposing, places them in a container with a plastic liner OR  If gloves are reusable, immerses them in a 0.5% chlorine solution for at least 10 minutes | | |  | |  |
|  | 13.9 | Performs hand hygiene after removing gloves | | |  | |  |
|  | 13.10 | Record relevant details (childbirth, AMTSL, placenta examination, newborn condition, episiotomy (if done)) on the mother’s record | | |  | |  |
|  | **Achieved: Yes/No (Circle the answer)** | | | | | |  |
| 14. The provider properly monitors the newborn in immediate postpartum period. (Verify by observation that the baby is monitored correctly in first two hours and findings are documented) and at least once perform detail examination within 12 hours of birth. | 14.1 | Tell the mother and her support person what is going to be done (examining the newborn), listen to her and respond attentively to her questions and concerns. | | |  | |  |
|  | 14.2 | Wash hands thoroughly with soap and water and dry with a clean, dry cloth or air dry. | | |  | |  |
|  | 14.3 | Place newborn on a clean, warm surface where the mother can see what will be done. | | |  | |  |
|  | 14.4 | Provider checks that the baby is warm,  If cold, takes axillary’s temperature and make sure the baby is kept warm by maintaining skin-to-skin contact or if skin-to-skin contact is not possible, re-wrap the baby, including the head, and place the baby under a heat source or in incubator | | |  | |  |
|  | 14.5 | Positions the head of the baby so that the neck is slightly extended | | |  | |  |
|  | 14.6 | Stimulates the baby by rubbing in the back | | |  | |  |
|  | 14.7 | Instruct that Baby’s vital signs are checked every 15 minutes in first hour of birth and then every 30 minutes the second hour | | |  | |  |
|  | 14.8 | Counter checks that the baby has no bleeding from cord | | |  | |  |
|  | 14.9 | | History (Ask/Listen) – or recollect information from mother or her accompanied person |  | |  | |
|  | 14.10. Check the mother’s record for other conditions/factors or ask her if she had: | | | | | | |
|  | 14.10.a | Eclampsia | | |  | |  |
|  | 14.10.b | Breech delivery | | |  | |  |
|  | 14.10.c | Delivery by vacuum extraction | | |  | |  |
|  | 14.11 | Ensures that the breastfeeding is initiated | | |  | |  |
|  | 14.12 | Observe the newborn at the breast, if s/he is ready to feed | | |  | |  |
|  | 14.13 | Ask mother to put the baby to breast to observer attachment, sucking and positioning. | | |  | |  |
|  | 14.14 Physical Examination ( Look/Feel) | | | | | | |
|  | 14.14.a | General appearance (alert or lethargic , cyanosed) | | |  | |  |
|  | 14.14.b | Cry (normal , irritable or high – pitched) | | |  | |  |
|  | 14.14.c | Breathing rate (normal range 30-40 | | |  | |  |
|  | 14.14.d | breaths/minute),grunting , chest indrawing | | |  | |  |
|  | 14.14.e | Heart rate(normal range 120-160/Minute) | | |  | |  |
|  | 14.14.f | Temperature (normal range 36.5-37.5) | | |  | |  |
|  | 14.15 | Weight the newborn. | | |  | |  |
|  | 14.16 | | Measure the head circumference |  | |  | |
|  | 14.17 | Check the skull contours and feel for the normal sutures and fontanelles | | |  | |  |
|  | 14.18 | Open the eyelids and check that eyes have a normal appearance and there are no signs of infection | | |  | |  |
|  | 14.19 | Check for any abnormalities of the face , especially for asymmetrical movement | | |  | |  |
|  | 14.20 | Examine the upper limbs and lower limbs: Check the skin , soft tissues and bones for abnormalities | | |  | |  |
|  | 14.21 | Examine the chest for symmetrical movement. | | |  | |  |
|  | 14.22 | Examine the umbilicus for bleeding and check that the tie is tightly applied. | | |  | |  |
|  | 14.23 | Examine the genitalia for abnormalities | | |  | |  |
|  | 14.24 | Check that the anus is patent. | | |  | |  |
|  | 14.25 | Examine the spine for abnormalities. | | |  | |  |
|  | 14.26 | Provide counseling about danger signs in the newborn period and what to do about them | | |  | |  |
|  | 14.27 | Ensures that the baby is not bathed within 3 days of birth. | | |  | |  |
|  | 14.28 | Wash hands thoroughly with soap and water and dry with a clean, dry cloth or air dry. | | |  | |  |
|  | 14.29 | Inform the mother of your findings and ask her if she has additional questions. | | |  | |  |
|  | 14.30 | Record all relevant findings from the physical examination. | | |  | |  |
|  | **Achieved: Yes/No (Circle the answer)** | | | | | |  |
| 15. The provider closely monitors the woman for at least two hours after the birth. (Observe the women after the delivery and at least once perform the detail examination within 12 hours of childbirth. | 15.1 | | Monitors the woman every 15 minutes in the first hour checking for vaginal bleeding |  | |  | |
|  | 15.2 | | Monitors the woman and the baby every 30 minutes in the second hour checking for vaginal bleeding |  | |  | |
|  | 15.3 | Asks the woman if she has urinated and encourages her to do so whenever she wishes | | |  | |  |
|  | 15.4 | Check the mother’s record or ask for her name, age, parity and the number of children she has. | | |  | |  |
|  | 15.5 | Ask the mother how she is feeling and whether she has had any problems since the birth of her baby. | | |  | |  |
|  | 15.6 | Ask the mother about breastfeeding and whether she has had any problems. | | |  | |  |
|  | 15.7 | Ask the mother about family planning, including method preference. | | |  | |  |
|  | 15.8 | Help the mother onto the examination table and place a pillow under her head and upper shoulders. | | |  | |  |
|  | 15.9 | Wash hands thoroughly. | | |  | |  |
|  | 15.10 | Explain each step of Physical Examination. | | |  | |  |
|  | 15.11 | Take the mother’s temperature and blood pressure. | | |  | |  |
|  | 15.12 | Check the mother’s conjunctiva and palms for pallor. | | |  | |  |
|  | 15.13 | Examine the breasts for engorgement and cracked/sore nipples. | | |  | |  |
|  | 15.14 | Examine the abdomen to check the uterus and detect tenderness. | | |  | |  |
|  | 15.15 | Examine legs for pain and tenderness. | | |  | |  |
|  | 15.16 | Performs initial management in the event of hemorrhage | | |  | |  |
|  | 15.17 | Put on new examination or high-level disinfected surgical gloves. | | |  | |  |
|  | 15.18 | Examine perineum and genitalia for signs of trauma and infection. | | |  | |  |
|  | 15.19 | Observe color, odor and amount of lochia. | | |  | |  |
|  | 15.20 | Immerse both gloved hands in 0.5% chlorine solution and remove gloves by turning them inside out  If disposing of gloves , place in leak proof container or plastic bag OR  If reusing surgical gloves, submerge in 0.5% chlorine solution for 10 minutes to decontaminate. | | |  | |  |
|  | 15.21 | Wash hands thoroughly with soap and water and dries | | |  | |  |
|  | 15.22 | | Dispense/order for the mother | | |  | |
|  | 15.22.a | Iron-folate tablets (3-months supply) | | |  | |  |
|  | 15.22.b | Vitamin A (based on need and country/local policy) | | |  | |  |
|  | 15.22.c | Provide tetanus immunization based on need. | | |  | |  |
|  | 15.23 | Provide counseling about danger signs in the postpartum period and what to do about them. | | |  | |  |
|  | 15.24 | | Provide counseling about: | | |  | |
|  | 15.24.a | Nutrition and iron supplementation | | |  | |  |
|  | 15.24.b | Rest | | |  | |  |
|  | 15.24.c | Hygiene | | |  | |  |
|  | 15.24.d | Not to have intercourse before 6 weeks | | |  | |  |
|  | 15.24.e | Safer sex and sexually transmitted infections | | |  | |  |
|  | 15.25 | | Provide counseling about family planning | | |  | |
|  | 15.25.a | Explain how lactational amenorrhea method (LAM) works | | |  | |  |
|  | 15.25.b | Help the mother choose an appropriate method of contraception if she does not want to use LAM | | |  | |  |
|  | 15.25.c | If the mother is not breastfeeding, explain the return of menstrual cycles and help her to choose an appropriate method of contraception | | |  | |  |
|  | 15.25.d | Provide method of choice and instructions for use | | |  | |  |
|  | 15.25.e | Discuss what to do if side effects are experienced | | |  | |  |
|  | 15.26 | Provide follow-up instructions | | |  | |  |
|  | 15.27 | Ask the mother if she has any further questions or concerns | | |  | |  |
|  | 15.28 | Thank the mother and tell her when she should come for her next postpartum visit, if necessary | | |  | |  |
|  | 15.29 | Records the information on woman’s clinical record | | |  | |  |
|  | **Achieved: Yes/No (Circle the answer)** | | | | | |  |
| 16. The provider properly performs resuscitation of the newborn. (If need resuscitation of the newborn then 17.1 observed the following otherwise skip this section) | 16.1 In the event of resuscitation with bag and mask | | | | | | |
|  | 16.1.a | Places the mask so it covers the baby’s chin, mouth, and nose | | |  | |  |
|  | 16.1.b | Ensures that an appropriate seal has been formed between mask, nose, mouth and chin | | |  | |  |
|  | 16.1.c | Ventilates 40 times per minute for 1 minute | | |  | |  |
|  | 16.1.d | Pauses and determines whether the baby is breathing spontaneously  (If the baby is breathing and there is no sign of respiratory difficulty (intercostal retractions or grunting), place the baby in skin-to-skin contact with mother) | | |  | |  |
|  | 16.2 If the baby does not begin to breathe or if breathing is less than 20/min minute or gasping: | | | | | | |
|  | 16.2.a | Continue artificial ventilation | | |  | |  |
|  | 16.2.b | Assesses the need for special care | | |  | |  |
|  | 16.2.c | Explains to the mother what is happening, if possible | | |  | |  |
|  | 16.3 If there is no breathing after 20 minutes of ventilation or gasping (type of breathing )for 30 minutes | | | | | | |
|  | 16.3.a | Suspends resuscitation | | |  | |  |
|  | 16.3.b | Records the time of death | | |  | |  |
|  | 16.4 | Provides emotional support to mother/parents and family members | | |  | |  |
|  | 16.5 | Record all actions taken on the woman’s clinical record | | |  | |  |
|  | 16.6 | Asks the mother whether she has any questions, and responds using easy-to-understand language | | |  | |  |
|  | 16.7 | Thank the mother for coming and tell her when she should come for her next postpartum visit, if necessary | | |  | |  |
|  | **Achieved: Yes/No (Circle the answer)** | | | | | |  |

|  | Total Number | Observe numbers | Achievement | Proportion |
| --- | --- | --- | --- | --- |
| 1. Standard / Components | 16 |  |  |  |
| 2. Activities | 258 |  |  |  |

1. **Procedure done by**

| **a. Designation of the provider** | **b. which part of the procedure done** |
| --- | --- |
| **1.** | **1.** |
| **2.** | **2.** |
| **3.** | **3.** |
| **4.** | **4.** |
| **5.** | **5.** |
| **6.** | **6.** |

**Code list for designation of the provider:** 01=Consultant/Specialist in Ob/Gyn, 02=MO/Assistant Register, 03=Consultant/Specialist in Anaesthesia, 04=Consultant/Specialist in Paediatrics, 05=SSN/SN, 06=FWV/Senior FWV, 07=HA/SACMO/ MA/ Paramedics, 08= FWA, 09= CHCP/CSBA/ Community volunteer, 10=Assistant Nurse/ Student nurse , 11= ANA/Nurse AID/FMA/ Aya/ Dai nurse/ OT boy, 12= MT, 13=Sweeper/Cleaner/MLSS/Ward boy/Driver,

14= Others (specify_________________________________________________)

1. **Particulars of the primary provider:**

| 1. Sex Male = 1, Female = 2 |  | 4. Years of service | ­­­­Yrs |
| --- | --- | --- | --- |
| 2. Designation |  | 5. Years of service in this facility | Yrs |
| 3. Professional qualification/ Training | a. | b. | c. |

**Code list for Qualification:** 01=FCPS/MCPS/DGO, 02=MBBS, 03=Post graduate training, 04= EOC training, 05=Basic training (FWV/SACMO/Paramedics), 06= Basic training (CHCP/HA), 07=Diploma /BSC in nursing, 08=Midwifery, 09=SBA/TBA/CSBA training, 10=Any other short training, 11=Study in nursing, 12= Others (specify________________________________________________________________________)

1. **Particulars of the Mother:** Collect information from the health care provider at the end of the observation

| 1. Age | Yrs | 2. Para (+Abortus/miscarriage) |  |
| --- | --- | --- | --- |
| 3. Gravida |  | 4. Gestational age | Weeks |
| 5. First pregnancy  Yes = 1 , No = 2 |  | 6. Multiple Pregnancy Yes = 1 , No = 2 |  |
| 7. Type of delivery NVD=1, CS=2, Miscarraige =3, Others ____________________________________________________=4 | | | |
| 8. Any high risk indicator | a. | b. | c. |

**(Gravida**indicates the number of times the mother has been pregnant, regardless of whether these pregnancies were carried to term. A current pregnancy, if any, is included in this count. **Para** indicates the number of >20 wks births (including viable and non-viable i.e. stillbirths). Pregnancies consisting of multiples, such as twins or triplets, count as ONE birth for the purpose of this notation. **Abortus**is the number of pregnancies that were lost for any reason, including induced abortions or miscarriages. The abortus term is sometimes dropped when no pregnancies have been lost. Stillbirths are not included.)

**Code list for High risk factor:** 01=Previous C/S, 02=Pre-eclampsia /Eclampsia, 03=Bad obstetric history, 04= Malpresentation, 05=Sub-fertility, 06=Oligo-hydramnios, 07= Post dated , 08=Incomplete abortion, 09=Fetal distress, 10=Obstructed labor,11= PROM/ Leaking membrane,12= Multiple pregnancy,13=Home trialed, 14=Other Medical problem,15=PV bleeding,16= others (specify_______________________)

| **Comments** |
| --- |
|  |

**Observation End Time: |___||___|:|___||___|**

Signature of the Observer: __________________________ **Date:** ___/___/2014

Signature of the Supervisor: __________________________ **Date:** ___/___/2014

Signature of the Data entry personnel: ________________________ **Date:** ___/___/2014
