# Supplementary material for: Quality of maternal and newborn healthcare services in two public hospitals of Bangladesh: identifying gaps and provisions for improvement
Source: BMC Pregnancy Childbirth. 2019 Dec 10;19:488. doi: 10.1186/s12884-019-2656-1 (PMC6905111; doi:10.1186/s12884-019-2656-1)
Supplement: Supplementary file 2 — Additional file 2. Checklist_ANC.docx (ANC Checklist). [file 12884_2019_2656_MOESM2_ESM.docx]

**Appendix V: Checklists to observe quality of care of MNH Cases attending the Health Facility**

**International Centre for Diarrhoeal Disease Research, Bangladesh (icddr,b)**

**AREA 2: ANTENATAL CARE**

**Facility Name: _____________________________________ Facility Type: ______________________________**

**District: _________________________________ Upazilla: _____________________**

**UFI of the facility:** |___|___|___|___|___|___|___|___|

**Place of observation: ____________________________________**

**Code list:** 01= OPD/EPI room, 02=Ward/Cabin, 03=ANC room, 04=Labor/Delivery room, 05=OT,

06=Nurse/ SACMO/CHCP Room, 07= Others (specify_______________________________)

**Assessment Type:** (BASELINE 🞎/PERIODIC🞎)

**Phase of Data collection:** Phase I 🞎/Phase II 🞎/Phase III 🞎

**Name of the Observer** ___________________________________

**Case no:** |___|___| **Patient no:** |___|___|___|___|

**Date:** ___/___/2014  **Observation Start Time: |___||___|:|___||___|**

**Operational definition:**

- **Done**: Performs the step or task according to the standard procedure or guidelines.
- **Not done:** Unable to perform the step or task according to the standard procedure or guidelines.
- **Not applicable**: Step or task not applicable for that particular patient during evaluation by observer.

| **PERFORMANCE STANDARDS** | **Sl no** | **VERIFICATION CRITERIA** | **Observation**  **[Done=1, Not done=0,**  **Not applicable=9** | **COMMENTS** |
| --- | --- | --- | --- | --- |
| 1. The person who receives the pregnant woman conducts a **rapid initial evaluation** at the first contact. | **1.1 Determine whether the provider/receptionist asks the pregnant woman upon her arrival in the facility whether she has or has had any medical problem** | | |  |
|  | 1.1a | Vaginal bleeding |  |  |
|  | 1.1.b | Respiratory difficulty |  |  |
|  | 1.1.c | Fever |  |  |
|  | 1.1.d | Severe headache/blurred vision |  |  |
|  | 1.1.e | Severe abdominal pain |  |  |
|  | 1.1.f | Convulsions/loss of consciousness |  |  |
|  | 1.2 | Assures immediate attention in the event of any of the above signs |  |  |
|  | **Achieved : Yes / No (Circle the answer)** | | |  |
| 1. The provider receives and treats the pregnant woman and her husband or companion cordially and respectfully. ( Observe during care of pregnant women whether the provider | 2.1 | Greets the woman and her husband or companion (if present) in a cordial manner |  |  |
|  | 2.2 | Introduces her/him self and speaks in easy to understand language for the client |  |  |
|  | 2.3 | Encourages the woman to ask her husband or companion to remain at her side, as appropriate |  |  |
|  | 2.4 | Maintains privacy during the provision of care |  |  |
|  | 2.5 | Explains to the woman and her companion what she/he is going to do and encourages her to ask questions |  |  |
|  | **Achieved : Yes / No (Circle the answer)** | | |  |
| 3.The provider obtains/reviews the obstetrical history.(Observe during the care provided to pregnant women whether the provider asks/reviews the following information) | 3.1 | Confirms woman’s name and age |  |  |
|  | 3.2 | Number and type of previous pregnancies (ectopic pregnancies, premature and large babies, still birth) including abortions |  |  |
|  | 3.3 | Outcome of previous pregnancies and dates of delivery, mode of delivery, sex of babies, neonatal death or any complications |  |  |
|  | 3.4 | History of menstrual and contraceptives |  |  |
|  | 3.5 | Date of last delivery |  |  |
|  | 3.6 | Date of the first day of her last menstrual period and regularity of menses |  |  |
|  | 3.7 | Calculates expected date of delivery (EDD) and gestational age |  |  |
|  | 3.8 | History of violence or abuse during pregnancy |  |  |
|  | 3.9 | Do she and her husband want to use a family planning method after this baby is born? If yes, ask for details. |  |  |
|  | **Achieved : Yes / No (Circle the answer)** | | |  |
| 4.The provider takes/reviews the medical history.( Observe during the care provided to pregnant woman whether the provider completes/reviews a medical history for the woman and records it) | 4.1 | Asks about and records any general health problems (i.e., headache, fever, joint pain, chronic diarrhea, weight loss, vaginal discharge, genital ulcers, etc.) |  |  |
|  | **4.2 Reviews in the card, if not present, asks about and records:** | | |  |
|  | 4.2.a | Full or partial tetanus toxoid immunization |  |  |
|  | 4.2.b | Allergies (medical or other) |  |  |
|  | 4.2.c | Current medications and/or herbal treatments |  |  |
|  | 4.3 | Any history of diabetes, tuberculosis, hypertension, heart diseases, urinary track infections/STI, malaria or others |  |  |
|  | 4.4 | Any history of surgical interventions (specify) |  |  |
|  | 4.5 | Any family history of diabetes, tuberculosis, hypertension, heart diseases or others |  |  |
|  | 4.6 | Current use of tobacco or other harmful substance |  |  |
|  | **Achieved : Yes / No (Circle the answer)** | | |  |
| 5. The provider properly conducts a physical and obstetric examination. (Observe during care of pregnant women whether the provider) | 5.1 | Explains each stage of the examination to the woman using easy-to-understand language |  |  |
|  | 5.2 | Ensures privacy |  |  |
|  | 5.3 | Adequate light |  |  |
|  | 5.4 | Asks woman to empty her bladder |  |  |
|  | 5.5 | Saves urine for testing |  |  |
|  | 5.6 | Prepare all the necessary instruments for physical examination |  |  |
|  | 5.7 | Helps the woman to climb up onto the examining table, and places a pillow under her head |  |  |
|  | **PHYSICAL EXAMINATION** | | |  |
|  | 5.8 | Washes hands with soap and water and dries them |  |  |
|  | 5.9 | Check Pulse |  |  |
|  | 5.10 | Check Blood pressure |  |  |
|  | 5.11 | Check Temperature (only if the woman complains of fever)  Check conjunctiva and complexion for anemia |  |  |
|  | 5.12 | Measure weight |  |  |
|  | 5.13 | Measure height |  |  |
|  | 5.14 | Check oedema |  |  |
|  | 5.15 | Check jaundice (check eye, skin colour) |  |  |
|  | 5.16 | Examination of breast |  |  |
|  | **OBSTETRIC EXAMINATION** | | |  |
|  | 5.17 | Measures fundal height |  |  |
|  | 5.18 | If after 20 weeks, listens to the fetal heart rate |  |  |
|  | 5.19 | If after 36 weeks, determines fundal height, presentation, if necessary fetal lie |  |  |
|  | 5.20 | Helps her to get down from the examining table |  |  |
|  | 5.21 | Washes hands with soap and water and dries them |  |  |
|  | 5.22 | Records all relevant finding in the woman’s ANC/PNC card |  |  |
|  | 5.23 | Informs woman on key findings |  |  |
|  | **Achieved : Yes / No (Circle the answer)** | | |  |
| 6. The provider properly conducts individualized care based on findings and protocols. (Observe during care of a pregnant woman whether the provider) | **When appropriate, conducts any needed lab evaluations based on exam, possibly including:** | | |  |
|  | 6.1 | Perform or advice for haemoglobin/complete Blood Count (CBC) |  |  |
|  | 6.2 | Perform or advice for urinalysis for sugar and protein |  |  |
|  | 6.3 | Perform or advice for syphilis screening (VDRL) |  |  |
|  | 6.4 | Perform or advice for blood grouping and Rh factor |  |  |
|  | 6.5 | Perform or advice for HIV (only after counseling and consent of the woman) |  |  |
|  | **Provides routine medications:** | | |  |
|  | 6.6 | Provides Ferrous Folate once daily in enough amounts to last until next visit (60 mg Iron and 400 mcg Folic acid) |  |  |
|  | 6.7 | If Hb is less than 7g% doubles the dose of iron |  |  |
|  | 6.8 | Explains side effects of taking iron folic acid tablets |  |  |
|  | 6.9 | Counsels about eating food rich in Iron and Vitamin C and to avoid tea, coffee and colas with iron |  |  |
|  | 6.10 | Give TT based on woman’s need, according to protocol |  |  |
|  | 6.11 | Provides specific advice and counselling to the woman and her husband or companion as needed (i.e., common discomfort, rest, safe sex, nutrition, hygiene and breast feeding) |  |  |
|  | **Achieved : Yes / No (Circle the answer)** | | |  |
| 7. The provider ensures that all women and their husbands/companions are prepared for a complication arising. (Observe during a visit with a woman in her second or third trimester, that the provider helps the client and her husband/partner develop an individual birth plan (IBP) | **7.1 Explains the benefits of giving birth with a skilled provider who knows how to treat complications**  **Counsels and help to develops a birth plan and emergency readiness plan with the woman, including all preparations for normal birth and plan in case of emergency:** | | |  |
|  | 7.1.a | Skilled provider and place of birth |  |  |
|  | 7.1.b | Signs and symptoms of labor and when she has to call SBA or go to the facility |  |  |
|  | 7.1.c | - Identifies a person as birth companion and another who will take care of home and other children, when woman delivers and in the immediate postpartum period |  |  |
|  | 7.1.d | Emergency transportation |  |  |
|  | 7.1.e | Provider asks her to identify a family member(s)/relative/friend as a blood donor |  |  |
|  | 7.1.f | Advises the woman and her family to keep a small amount of money for emergency |  |  |
|  | 7.1.g | Items for clean and safe birth |  |  |
|  | 7.1.h | Decision-making person in case complication occurs at home |  |  |
|  | **7.2 Explains danger sign and symptoms of pregnancy**  **Danger signs and symptoms:**  **- Where to go, in case of any danger sign** | | |  |
|  | 7.2.a | Vaginal bleeding |  |  |
|  | 7.2.b | Fever |  |  |
|  | 7.2.c | Severe headache/Blurred vision |  |  |
|  | 7.2.d | Prolong labour / sever abdominal pain/prolapse of cord |  |  |
|  | 7.2.e | Convulsions/loss of consciousness |  |  |
|  | **Achieved : Yes / No (Circle the answer)** | | |  |
| 8. The provider evaluates the care and plans the return visit with the pregnant woman and her husband/companion.( Observes whether the provider) | 8.1 | Asks the woman and her husband/companion, if present, to repeat the most important points of the counselling |  |  |
|  | 8.2 | Asks about, and responds to, any question that the woman and/or her husband/companion asks |  |  |
|  | 8.3 | Sets a date for the next visit according to findings and recommends minimum of four antenatal visits |  |  |
|  | 8.4 | Tells the woman and her husband/companion that she must come immediately if she has any danger signs and symptoms or go to a comprehensive health facility |  |  |
|  | 8.5 | Thanks the woman for coming |  |  |
|  | **Achieved : Yes / No (Circle the answer)** | | |  |

|  | Total Number | Observe numbers | Achievement | Proportion |
| --- | --- | --- | --- | --- |
| 1. Standard / Components | 8 |  |  |  |
| 2. Activities | 81 |  |  |  |

**9. Procedure done by**

| **a. Designation of the provider** | **b. which part of the procedure done** |
| --- | --- |
| **1.** | **1.** |
| **2.** | **2.** |
| **3.** | **3.** |
| **4.** | **4.** |
| **5.** | **5.** |
| **6.** | **6.** |

**Code list for designation of the provider:** 01=Consultant/Specialist in Ob/Gyn, 02=MO/Assistant Register, 03=Consultant/Specialist in Anaesthesia, 04=Consultant/Specialist in Paediatrics, 05=SSN/SN, 06=FWV/Senior FWV, 07=HA/SACMO/ MA/ Paramedics, 08= FWA, 09= CHCP/CSBA/ Community volunteer, 10=Assistant Nurse/ Student nurse , 11= ANA/Nurse AID/FMA/ Aya/ Dai nurse/ OT boy, 12= MT, 13=Sweeper/Cleaner/MLSS/Ward boy/Driver,

14= Others (specify_________________________________________________)

1. **Particulars of the primary provider:**

| 1. Sex Male = 1, Female = 2 |  | 4. Years of service | ­­­­Yrs |
| --- | --- | --- | --- |
| 2. Designation |  | 5. Years of service in this facility | Yrs |
| 3. Professional qualification/ Training | a. | b. | c. |

**Code list for Qualification:** 01=FCPS/MCPS/DGO, 02=MBBS, 03=Post graduate training, 04= EOC training, 05=Basic training (FWV/SACMO/Paramedics), 06= Basic training (CHCP/HA), 07=Diploma /BSC in nursing, 08=Midwifery, 09=SBA/TBA/CSBA training, 10=Any other short training, 11=Study in nursing, 12= Others (specify________________________________________________________________________)

1. **Particulars of the Mother:** Collect information from the health care provider at the end of the observation

| 1. Age | Yrs | 2. Para (+Abortus/miscarriage) |  |
| --- | --- | --- | --- |
| 3. Gravida |  | 4. Gestational age | Weeks |
| 5. First pregnancy  Yes = 1 , No = 2 |  | 6. Multiple Pregnancy Yes = 1 , No = 2 |  |
| 7. Type of delivery NVD=1, CS=2, Miscarraige =3, Others ____________________________________________________=4 | | | |
| 8. Any high risk indicator | a. | b. | c. |

**(Gravida**indicates the number of times the mother has been pregnant, regardless of whether these pregnancies were carried to term. A current pregnancy, if any, is included in this count. **Para** indicates the number of >20 wks births (including viable and non-viable i.e. stillbirths). Pregnancies consisting of multiples, such as twins or triplets, count as ONE birth for the purpose of this notation. **Abortus**is the number of pregnancies that were lost for any reason, including induced abortions or miscarriages. The abortus term is sometimes dropped when no pregnancies have been lost. Stillbirths are not included.)

**Code list for High risk factor:** 01=Previous C/S, 02=Pre-eclampsia /Eclampsia, 03=Bad obstetric history, 04= Malpresentation, 05=Sub-fertility, 06=Oligo-hydramnios, 07= Post dated , 08=Incomplete abortion, 09=Fetal distress, 10=Obstructed labor,11= PROM/ Leaking membrane,12= Multiple pregnancy,13=Home trialed, 14=Other Medical problem,15=PV bleeding,16= others (specify_______________________)

| **Comments** |
| --- |
|  |

**Observation End Time: |___||___|:|___||___|**

Signature of the Observer: __________________________ **Date:** ___/___/2014

Signature of the Supervisor: __________________________ **Date:** ___/___/2014

Signature of the Data entry personnel: ________________________ **Date:** ___/___/2014
