# Supplementary material for: Quality of maternal and newborn healthcare services in two public hospitals of Bangladesh: identifying gaps and provisions for improvement
Source: BMC Pregnancy Childbirth. 2019 Dec 10;19:488. doi: 10.1186/s12884-019-2656-1 (PMC6905111; doi:10.1186/s12884-019-2656-1)
Supplement: Supplementary file 3 — Additional file 3. Checklist_Blood Transfustion.docx (Blood transfussion checklist). [file 12884_2019_2656_MOESM3_ESM.docx]

**Appendix V: Checklists to observe quality of care of MNH Cases attending the Health Facility**

**International Centre for Diarrhoeal Disease Research, Bangladesh (icddr,b)**

**AREA 8: BLOOD TRANSFUSION**

**Facility Name: _____________________________________ Facility Type: ______________________________**

**District: _________________________________ Upazilla: _____________________**

**UFI of the facility:** |___|___|___|___|___|___|___|___|

**Place of observation: ____________________________________**

**Code list:** 01= OPD/EPI room, 02=Ward/Cabin, 03=ANC room, 04=Labor/Delivery room, 05=OT,

06=Nurse/ SACMO/CHCP Room, 07= Others (specify_______________________________)

**Assessment Type:** (BASELINE 🞎/PERIODIC🞎)

**Phase of Data collection:** Phase I 🞎/Phase II 🞎/Phase III 🞎

**Name of the Observer** ___________________________________

**Case no:** |___|___| **Patient no:** |___|___|___|___|

**Date:** ___/___/2014  **Observation Start Time: |___||___|:|___||___|**

**Operational definition:**

- **Done**: Performs the step or task according to the standard procedure or guidelines.
- **Not done:** Unable to perform the step or task according to the standard procedure or guidelines.
- **Not applicable**: Step or task not applicable for that particular patient during evaluation by observer.

| **PERFORMANCE STANDARDS** |  | **VERIFICATION CRITERIA** | **Observation**  **[Done=1, Not done=0,**  **Not applicable=9** | **COMMENTS** |
| --- | --- | --- | --- | --- |
| 1. The provider treats the pregnant woman cordial manner. (Observe one woman for blood transfusion) | 1.1 | Ensures that she/he speaks in easy to understand language with the client |  |  |
|  | 1.2 | Greets the woman and her husband or companion in a cordial manner and introduces her/himself |  |  |
|  | 1.3 | Maintain privacy |  |  |
|  | 1.4 | Responds to questions and concerns |  |  |
|  | **Achieved : Yes / No (Circle the answer)** | | |  |
| 2. The provider properly reviews all the clinical finding and assesses the donor whether he/ she fit for blood Donation. (Observe blood collection at the **blood bank or laboratory**) | 2.1 | Asks and check papers of donor blood grouping & Rh typing. |  |  |
|  | 2.2 | Look for any apparent illness |  |  |
|  | 2.3 | Check weight, pulse, blood pressure, anemia |  |  |
|  | 2.4 | Go through cross matching & screening paper |  |  |
|  | **Achieved : Yes / No (Circle the answer)** | | |  |
| 3. The provider properly conducts the blood collection procedure task. (Observe blood collection at the **blood bank or laboratory**) | 3.1 | Maintain strict asepsis during collection & blood storage |  |  |
|  | 3.2 | Use sterile needle |  |  |
|  | 3.3 | Use standard blood bag filled with anticoagulant |  |  |
|  | 3.4 | Assurance the donor |  |  |
|  | 3.5 | Record keeping , marking particulars on bag |  |  |
|  | 3.6 | Maintain temperature of storage site |  |  |
|  | **Achieved : Yes / No (Circle the answer)** | | |  |
| 4. The provider properly conducts blood transfusion procedure. (Observe one woman for blood transfusion for any maternal complication) | 4.1 | Check blood grouping, cross matching and screening papers |  |  |
|  | 4.2 | Confirm indication of transfusion |  |  |
|  | 4.3 | Give transfusion order |  |  |
|  | 4.4 | Check transfusion order |  |  |
|  | 4.5 | Use sterilized blood transfusion set |  |  |
|  | 4.6 | Transfuse blood |  |  |
|  | 4.7 | Monitor patient during transfusion |  |  |
|  | 4.8 | Inform duty doctor if there is any complication |  |  |
|  | 4.9 | Proper disposal of bag and needle |  |  |
|  | 4.10 | Record all findings |  |  |
|  |  | | |  |

|  | Total Number | Observe numbers | Achievement | Proportion |
| --- | --- | --- | --- | --- |
| 1. Standard / Components | 4 |  |  |  |
| 2. Activities | 24 |  |  |  |

1. **Procedure done by**

| **a. Designation of the provider** | **b. which part of the procedure done** |
| --- | --- |
| **1.** | **1.** |
| **2.** | **2.** |
| **3.** | **3.** |
| **4.** | **4.** |
| **5.** | **5.** |
| **6.** | **6.** |

**Code list for designation of the provider:** 01=Consultant/Specialist in Ob/Gyn, 02=MO/Assistant Register, 03=Consultant/Specialist in Anaesthesia, 04=Consultant/Specialist in Paediatrics, 05=SSN/SN, 06=FWV/Senior FWV, 07=HA/SACMO/ MA/ Paramedics, 08= FWA, 09= CHCP/CSBA/ Community volunteer, 10=Assistant Nurse/ Student nurse , 11= ANA/Nurse AID/FMA/ Aya/ Dai nurse/ OT boy, 12= MT, 13=Sweeper/Cleaner/MLSS/Ward boy/Driver,

14= Others (specify_________________________________________________)

1. **Particulars of the primary provider:**

| 1. Sex Male = 1, Female = 2 |  | 4. Years of service | ­­­­Yrs |
| --- | --- | --- | --- |
| 2. Designation |  | 5. Years of service in this facility | Yrs |
| 3. Professional qualification/ Training | a. | b. | c. |

**Code list for Qualification:** 01=FCPS/MCPS/DGO, 02=MBBS, 03=Post graduate training, 04= EOC training, 05=Basic training (FWV/SACMO/Paramedics), 06= Basic training (CHCP/HA), 07=Diploma /BSC in nursing, 08=Midwifery, 09=SBA/TBA/CSBA training, 10=Any other short training, 11=Study in nursing, 12= Others (specify________________________________________________________________________)

1. **Particulars of the Mother:** Collect information from the health care provider at the end of the observation

| 1. Age | Yrs | 2. Para (+Abortus/miscarriage) |  |
| --- | --- | --- | --- |
| 3. Gravida |  | 4. Gestational age | Weeks |
| 5. First pregnancy  Yes = 1 , No = 2 |  | 6. Multiple Pregnancy Yes = 1 , No = 2 |  |
| 7. Type of delivery NVD=1, CS=2, Miscarriage =3, Others ____________________________________________________=4 | | | |
| 8. Any high risk indicator | a. | b. | c. |

**(Gravida**indicates the number of times the mother has been pregnant, regardless of whether these pregnancies were carried to term. A current pregnancy, if any, is included in this count. **Para** indicates the number of >20 wks births (including viable and non-viable i.e. stillbirths). Pregnancies consisting of multiples, such as twins or triplets, count as ONE birth for the purpose of this notation. **Abortus**is the number of pregnancies that were lost for any reason, including induced abortions or miscarriages. The abortus term is sometimes dropped when no pregnancies have been lost. Stillbirths are not included.)

**Code list for High risk factor:** 01=Previous C/S, 02=Pre-eclampsia /Eclampsia, 03=Bad obstetric history, 04= Malpresentation, 05=Sub-fertility, 06=Oligo-hydramnios, 07= Post dated , 08=Incomplete abortion, 09=Fetal distress, 10=Obstructed labor,11= PROM/ Leaking membrane,12= Multiple pregnancy,13=Home trialed, 14=Other Medical problem,15=PV bleeding,16= others (specify_______________________)

| 1. **Comments** |
| --- |
|  |

**Observation End Time: |___||___|:|___||___|**

Signature of the Observer: __________________________ **Date:** ___/___/2014

Signature of the Supervisor: __________________________ **Date:** ___/___/2014

Signature of the Data entry personnel: ________________________ **Date:** ___/___/2014
