# Supplementary material for: Quality of maternal and newborn healthcare services in two public hospitals of Bangladesh: identifying gaps and provisions for improvement
Source: BMC Pregnancy Childbirth. 2019 Dec 10;19:488. doi: 10.1186/s12884-019-2656-1 (PMC6905111; doi:10.1186/s12884-019-2656-1)
Supplement: Supplementary file 4 — Additional file 4. Checklist_Caesarean Section Delivery.doc (Cesarean delivery checklist). [file 12884_2019_2656_MOESM4_ESM.doc]

**Appendix V: Checklists to observe quality of care of MNH Cases attending the Health Facility**

**International Centre for Diarrhoeal Disease Research, Bangladesh (icddr,b)**

**AREA 6: COMPREHENSIVE EMERGENCE OBSTETRIC CARE - CAESAREAN SECTION**

**Facility Name: _____________________________________ Facility Type: ______________________________**

**District: _________________________________ Upazilla: _____________________**

**UFI of the facility:** |___|___|___|___|___|___|___|___|

**Place of observation: ____________________________________**

**Code list:** 01= OPD/EPI room, 02=Ward/Cabin, 03=ANC room, 04=Labor/Delivery room, 05=OT,

06=Nurse/ SACMO/CHCP Room, 07= Others (specify_______________________________)

**ASSESSMENT TYPE:** (BASELINE 🞎/PERIODIC¨)

**Phase of Data collection:** Phase I 🞎/Phase II 🞎/Phase III 🞎

**Name of the Observer** ___________________________________

**Case no:** |___|___| **Patient no:** |___|___|___|___|

**Date:** ___/___/2014  **Observation Start Time: |___||___|:|___||___|**

**Operational definition:**

- **Done**: Performs the step or task according to the standard procedure or guidelines.
- **Not done:** Unable to perform the step or task according to the standard procedure or guidelines.
- **Not applicable**: Step or task not applicable for that particular patient during evaluation by observer.

| **PERFORMANCE**  **STANDARDS** | | **No** | | **VERIFICATION CRITERIA** | | **Done=1,**  **Not done=0,**  **Not applicable=9** | **COMMENTS** | |
| --- | --- | --- | --- | --- | --- | --- | --- | --- |
| 1. The provider provides specific information about cesarean section delivery for the woman and her company. | | 1.1 | | Maintains privacy and confidentiality for the client | |  |  | |
|  |  | 1.2 | | Uses the client’s name as appropriate for the setting | |  |  |  |
|  |  | 1.3 | | Includes client’s companion/family in important discussions with client’s consent and where appropriate | |  |  |  |
|  |  | 1.4 | | Speaks respectfully and professionally with the patient | |  |  |  |
|  |  | 1.5 | | Explain for the woman and her accompany (if present) about benefits and risks of caesarean section and help her to understands the nature of the procedure: | |  |  |  |
|  |  | 1.6 | | Explains to the client why caesarean section is recommended for delivery of her baby (in simple statement) | |  |  |  |
|  |  | 1.7 | | Provide a general description about how cesarean section is performed including type of anesthesia and type of skin incision | |  |  |  |
|  |  | 1.8 | | Explain general risks of procedure | |  |  |  |
|  |  | 1.9 | | Reviews other disadvantages: May require repeat caesarean section for future deliveries, scarring on abdomen | |  |  |  |
|  |  | 1.10 | | Provide adequate PPFP counselling, including PPIUCD, and ensure the provision of the method as per client requests (as appropriate) | |  |  |  |
|  |  | 1.11 | | Encourage the client to ask question about treatment and answers client’s questions | |  |  |  |
|  |  | 1.12 | | Confirms that the client has freely consent with the caesarean section (Where appropriate and possible, obtains signed written consent from client to proceed with caesarean section) | |  |  |  |
|  |  | 1.13 | | Review the clinical history, perform physical and obstetric examination and confirms the foetus conditions and presentation | |  |  | |
|  |  | 1.14 | | Documents in medical record indication of caesarean section | |  |  | |
|  |  | **Achieved: Yes/No (Circle the answer)** | | | | |  | |
| 1. The provider requests and analysis the complementary laboratory exam of candidate for C-Section. | | 2.1 | | Examine the woman, assess her condition and check her vital signs | |  |  | |
|  |  | 2.2 | | Obtain blood for Haemoglobin ,clotting time, Rh factor, blood group and Cross match | |  |  | |
|  |  | 2.3 | | One unit blood and another donor should be kept ready | |  |  |  |
|  |  | 2.4 | | Fasting blood sugar (large baby of has history of Diabetes or suspect diabetes) | |  |  |  |
|  |  | 2.5 | | Routine Urine exam (Albumin, Glucose) | |  |  |  |
|  |  | **Achieved: Yes/No (Circle the answer)** | | | | |  |  |
| 1. The operate theatre (OT) and providers are prepared for the C section. (Pre-procedure tasks) | | 3.1 | | Operating table appropriate for caesarean delivery, with stirrups where available | |  |  | |
|  |  | 3.2 | | Operation light are available | |  |  | |
|  |  | 3.3 | | The temperature of operation theatre is appropriated | |  |  |  |
|  |  | 3.4 | | The surgeon and assistants use Protective glasses, cap, masks, apron, enclosed shoes | |  |  |  |
|  |  | 3.5 | | Perform a surgical hand scrub and put on sterile surgical gown and wear sterile surgical gloves | |  |  |  |
|  |  | 3.6 | | Ensure that those instruments and supplies are available and arrange them on a trolley. | |  |  |  |
|  |  | 3.7 | | Conduct an instrument and swab count and ask an assistant to note on board. | |  |  |  |
|  |  | 3.8 | | Parenteral antibiotics for surgical prophylaxis | |  |  |  |
|  |  | 3.9 | | Measure foetal heart sound | |  |  |  |
|  |  | 3.10 | | Measure vital signs of the patient | |  |  |  |
|  |  | 3.11 | | Drugs intended for use are drawn up into labeled syringes | |  |  |  |
|  |  | **Achieved: Yes/No (Circle the answer)** | | | | |  |  |
| 1. The providers help patient to be prepared for C-section. | | 4.1 | | Helps the client put on a gown and cap | |  |  | |
|  |  | 4.2 | | Inserts an IV line and infuses IV fluids (lactated Ringer solution or saline with 5% dextrose | |  |  | |
|  |  | 4.3 | | Using sterile technique catheterizes the bladder and attaches to gravity drainage bag | |  |  |  |
|  |  | 4.4 | | Places patient in dorsal supine position on the operating table | |  |  |  |
|  |  | 4.5 | | Tilt operating table to the left or place a pillow under the mother’s right lower back | |  |  |  |
|  |  | 4.6 | | Listens to the fetal heart rate and records before beginning surgery | |  |  |  |
|  |  | 4.7 | | Places an external monitor and monitors for the patient’s blood pressure, pulse and oxygen saturation | |  |  |  |
|  |  | **Achieved: Yes/No (Circle the answer)** | | | | |  |  |
| 5. The anaesthetist conducts general anaesthesia properly. | | 5.1 | | Decide the type of anaesthesia based on situation (Urgency and patient condition): Spinal anaesthesia (Sub arachnoids injection of drug); General anaesthesia with intubation; Epidural anaesthesia; Local infiltration (in rare case to be performed by the surgeon) | |  |  | |
|  |  | 5.2 | | Performs hand hygiene and puts sterile/HLD gloves on both hands | |  |  | |
|  |  | 5.3 | | Correctly administers the selected type of anaesthesia | |  |  |  |
|  |  | 5.4 | | Assess vital sign and level of anaesthesia and allow the surgeon to start C‐section procedure | |  |  |  |
|  |  | 5.5 | | Maintains continuous monitoring of the patient’s blood pressure, pulse rate,  respiratory rate, oxygen saturation and medications during the evolution of the C‐section | |  |  |  |
|  |  | 5.6 | | Communicates as needed with the surgeon and patient | |  |  |  |
|  |  | 5.7 | | Ensures that client has adequate anaesthesia throughout caesarean section  procedure | |  |  |  |
|  |  | 5.8 | | Records findings and procedures on woman’s clinical record | |  |  |  |
|  |  | **Achieved: Yes/No (Circle the answer)** | | | | |  |  |
| 6.The provider correctly opens the abdominal layers for C-section (laparotomy) | | 6.1 | | Decides about the type and performs the incision | |  |  | |
|  |  | 6.2 | | Clamps and ties or cauterizes arterial and venous bleeding points as encountered | |  |  |  |
|  |  | 6.3 | | Incises the fascia with a scalpel and extends incision with scissors to accommodate anticipated size of newborn head and body | |  |  |  |
|  |  | 6.4 | | For pfannenstiel incision, clamps facial edges, lifts and then separates fascia sharply from underlying rectus muscle using mayo or other operative scissors, superiorly and inferiorly | |  |  |  |
|  |  | 6.5 | | Separates the rectus muscle as needed for exposure | |  |  |  |
|  |  | 6.6 | | Lifts the peritoneum with forceps and ensures that it is free of loops of bowel and well above the bladder dome | |  |  |  |
|  |  | 6.7 | | Opens the peritoneum with a scissors and extends peritoneal incision to accommodate anticipated size of newborn head and body | |  |  |  |
|  |  | 6.8 | | Inspect and palpate the uterus to determine incision site, and make sure there is no adhesion and abnormality, Identifies the midline of the uterus by checking position of round ligaments or uterine vessels. | |  |  |  |
|  |  | **Achieved: Yes/No (Circle the answer)** | | | | |  |  |
| 1. The provider correctly opens the   Uterus (hysterotomy). | | 7.1 | | Places a bladder retractor over the pubic bone | |  |  | |
|  |  | 7.2 | | Uses forceps to pick up the loose peritoneum covering the anterior surface of the LUS and incises with scissors | |  |  |  |
|  |  | 7.3 | | Extends the incision by placing scissors between the uterus and the loose peritoneum and cuts about 3 cm on each side in transverse fashion. | |  |  |  |
|  |  | 7.4 | | Pushes or sharply dissects the bladder downward off the lower uterine segment where possible | |  |  |  |
|  |  | 7.5 | | Replaces the bladder retractor over the pubic bone to retract the bladder downward and away from the LUS and confirms presentation and positioning of the foetus by manual palpation of the uterus | |  |  |  |
|  |  | 7.6 | | Using a scalpel, makes a 3 cm midline transverse incision in the lower uterine segment and cuts down to the level of the amniotic membranes. | |  |  |  |
|  |  | 7.7 | | If the lower uterine segment cannot be identified and/ or if surgical access is a problem, makes a 3 cm midline vertical uterine incision in the body of the uterus and cuts down to the level of the membranes. (High vertical or classical hysterotomy | |  |  |  |
|  |  | 7.8 | | Widens the incision manually (thin LUS) or with scissors staying well away from the uterine vessels bilaterally and from the bladder inferiorly (the uterine incision should be big enough to deliver the head and body of the baby | |  |  |  |
|  |  | 7.9 | | If the amniotic membranes are intact, ruptures them and informs newborn care person/team of color and quantity | |  |  |  |
|  |  | **Achieved: Yes/No (Circle the answer)** | | | | |  |  |
| 1. The provider correctly delivers the baby. | | 8.1 | | Places one hand inside the uterine cavity between the uterus and the presenting fetal part | |  |  | |
|  |  | 8.2 | | Performs the procedures to deliver the baby according to the fetus presentation | |  |  |  |
|  |  | 8.3 | | After delivery of the newborn, asks anesthesia or an assistant to give oxytocin 20 units in one litter IV fluid (normal saline or ringer’s lactate) at 60 drops per minute for 2 hours | |  |  |  |
|  |  | 8.4 | | Doubly clamps the umbilical cord and cuts in between the clamps | |  |  |  |
|  |  | 8.5 | | Hands newborn to newborn care person/team while maintaining sterility of gloves and gown | |  |  |  |
|  |  | 8.6 | | The newborn care person/team determine APGAR score at one minute and five minutes | |  |  |  |
|  |  | 8.7 | | After clamping the cord, asks anaesthesia or an assistant to give a single dose of prophylactic antibiotics. | |  |  |  |
|  |  | **Achieved: Yes/No (Circle the answer)** | | | | |  |  |
| 1. The provider correctly delivers the placenta and explores the peritoneal cavity. | | 9.1 | | Maintains gentle traction on the cord and massages the uterus either directly or through the abdominal wall. | |  |  | |
|  |  | 9.2 | | Delivers the placenta by manual extraction or with uterine massage | |  |  |  |
|  |  | 9.3 | | Massages the uterus to expel blood and clots and as needed for uterine atony | |  |  |  |
|  |  | 9.4 | | Inspects placenta for completeness or any abnormalities and the umbilical cord to documents number of cord vessels | |  |  |  |
|  |  | 9.5 | | Palpates the inside of the uterus (to rule out posterior or lateral wall rupture, to ensure normal uterine anatomy and to document complete removal of placenta) and inspects both annexes to ensure normal tubal and ovarian anatomy | |  |  |  |
|  |  | 9.6 | | If uterine tone is inadequate despite uterine massage and IV oxytocin and if client’s BP is <160/100, asks anesthesia or an assistant to administer ergometrine 0.2 mgs IM. | |  |  |  |
|  |  | 9.7 | | If patient is febrile, performs gentle pelvic and abdominal exploration to ensure normal appendix and to rule out abscess formation. | |  |  |  |
|  |  | **Achieved: Yes/No (Circle the answer)** | | | | |  |  |
| 1. The provider correctly closes the uterus after C-section delivery. | | 10.1 | | Grasps with clamps (Allis/ring) the corners of the uterine incision (lateral, for transverse and grasps with ring clamps aspects of the incision (the superior and inferior for transverse | |  |  | |
|  |  | 10.2 | | While placing clamp, checks inferiorly to makes sure hysterotomy is separate from the bladder | |  |  |  |
|  |  | 10.3 | | Looks carefully for any extension of the uterine incision either laterally or vertically into the cervix/upper vagina | |  |  |  |
|  |  | 10.4 | | Repairs the hysterotomy with 0 chromic catgut (or polyglycolic) suture. Takes care to avoid including the decidua in the closure. | |  |  |  |
|  |  | 10.5 | | Ensures that there is no further bleeding by carefully inspecting the closure line (s) with placement of additional figure of eight haemostatic sutures as necessary. | |  |  |  |
|  |  | 10.6 | | Checks the bladder for injury and repairs injury if necessary | |  |  |  |
|  |  | 10.7 | | Rechecks the uterus to make sure it is firm | |  |  |  |
|  |  | 10.8 | | Evacuates blood and clots from peritoneal cavity manually or with suction (where available) being careful to avoid injury to the bowel | |  |  |  |
|  |  | 10.9 | | Requests repeat sponge and instrument count by surgical assistant | |  |  |  |
|  |  | **Achieved: Yes/No (Circle the answer)** | | | | |  |  |
| 1. The provider correctly performs closure of the abdominal wall after cesarean section delivery. | | 11.1 | | After checking carefully for sub facial bleeders, closes the abdominal wall fascia with continuous or interrupted suture. | |  |  | |
|  |  | 11.2 | | Closes the fat layer, if unusually thick or if skin incision splayed, with an  interrupted plain suture. | |  |  |  |
|  |  | 11.3 | | Checks all layers for hemeostasis before completing closure and during closure  process. Repairs arterial and venous bleeders with suture or cautery (where  available) | |  |  |  |
|  |  | 11.4 | | Closes the skin with interrupted mattress sutures about 2 cm apart, using a cutting needle and 3‐0 nylon or silk. | |  |  |  |
|  |  | 11.5 | | Inspects skin closure and ensures there is no persistent oozing, cleans the wound with sterile gauze moistened in sterile water and gently dries with sterile gauze | |  |  |  |
|  |  | 11.6 | | Applies a sterile dressing and covers with water‐occlusive covering (where available) | |  |  |  |
|  |  | **Achieved: Yes/No (Circle the answer)** | | | | |  |  |
| 1. The provider/ assistant completes all post-procedure tasks. | | 12.1 | | Checks bladder gravity bag and record amount and color of urine | |  |  | |
|  |  | 12.2 | | Removes scalpel blade from knife handle by using artery forceps | |  |  |  |
|  |  | 12.3 | | Disposes of blade (s), needles and syringes in sharps container. Does not recap needle before disposal | |  |  |  |
|  |  | 12.4 | | Disposes of waste materials in a leak proof container or plastic container | |  |  |  |
|  |  | 12.5 | | Places all instruments in 0.5% chlorine solution for decontamination for 10  minutes | |  |  |  |
|  |  | 12.6 | | Removes PPE (personal protective equipment) in proper sequence and discards in a leak proof container or plastic bag if disposing or decontaminates them in 0.5% chlorine solution if reusing. | |  |  |  |
|  |  | 12.7 | | Uses alcoholic hand rub or washes hands thoroughly with soap and water for  10‐ 15 sec | |  |  |  |
|  |  | 12.8 | | Writes operation note and postoperative management instructions. | |  |  |  |
|  |  | **Achieved: Yes/No (Circle the answer)** | | | | |  |  |
| 1. The providers correctly monitors postoperative course and ensures postnatal care for the client. | | 13.1 | | Monitor and record pulse, blood pressure, respiration rate, wound/ vaginal bleeding, client pain level, mental status, urine output, dressing and contraction of uterus | |  |  | |
|  |  | 13.2 | | Administers oxygen and medications as necessary (Oxytocine and analgesic) | |  |  |  |
|  |  | 13.3 | | Directs bladder catheter and gravity drainage be continued with removal 12‐24 hours post operative for normal urine output and no hematuria | |  |  |  |
|  |  | **13.4 The anesthetist permit the C‐ section patient to transfer in recovery room observing the following criteria on the:** | | | | |  |  |
|  |  | 13.4.a | | Patient awake, opens eyes, can lift head on command, extubated (as appropriate), with stable blood pressure and pulse, breathes quietly and comfortably, and with not hypoxic | |  |  |  |
|  |  | 13.4.b | | Appropriate pain management has been prescribed and safely established | |  |  |  |
|  |  | 13.4.c | | Adequate urine output | |  |  |  |
|  |  | **13.5 Midwife or duty nurse checks patient for deteriorated condition:** | | | | |  |  |
|  |  | 13.5.a | | Airway obstruction, Hypoxia | |  |  |  |
|  |  | 13.5.b | | Hemorrhage: internal or external | |  |  |  |
|  |  | 13.5.c | | Hypotension and/or hypertension | |  |  |  |
|  |  | 13.5.d | | Postoperative pain | |  |  |  |
|  |  | 13.5.e | | Shivering, hypothermia | |  |  |  |
|  |  | 13.5.f | | Vomiting, aspiration | |  |  |  |
|  |  | 13.5.g | | Falling on the floor/loss of consciousness, Residual narcosis | |  |  |  |
|  |  | 13.6 | | Ensure provision of breastfeeding as soon as possible and support and education including role of maternal nutrition and vitamin supplementation | |  |  |  |
|  |  | 13.7 | | Ensure/reinforce provision of adequate PPFP counseling and method as per client requests | |  |  |  |
|  |  | 13.8 | | Records relevant information about the services provided in the patient’s chart | |  |  |  |
|  |  | **Achieved: Yes/No (Circle the answer)** | | | | |  |  |
| 14. The provider properly monitors the newborn in immediate postpartum period. (Verify by observation that the baby is monitored correctly in first two hours and findings are documented) and at least once perform detail examination within 12 hours of birth. | | 14.1 | | | Tell the mother and her support person what is going to be done (examining the newborn), listen to her and respond attentively to her questions and concerns. |  |  | |
|  |  | 14.2 | | | Wash hands thoroughly with soap and water and dry with a clean, dry cloth or air dry. |  |  |  |
|  |  | 14.3 | | | Place newborn on a clean, warm surface where the mother can see what will be done. |  |  |  |
|  |  | 14.4 | | | Provider checks that the baby is warm,  If cold, takes axillary’s temperature and make sure the baby is kept warm by maintaining skin-to-skin contact or if skin-to-skin contact is not possible, re-wrap the baby, including the head, and place the baby under a heat source or in incubator |  |  |  |
|  |  | 14.5 | | | Positions the head of the baby so that the neck is slightly extended |  |  |  |
|  |  | 14.6 | | | Stimulates the baby by rubbing in the back |  |  |  |
|  |  | 14.7 | | | Instruct that Baby’s vital signs are checked every 15 minutes in first hour of birth and then every 30 minutes the second hour |  |  |  |
|  |  | 14.8 | | | Counter checks that the baby has no bleeding from cord |  |  |  |
|  |  | 14.9 | | | History (Ask/Listen) – or recollect information from mother or her accompanied person |  |  |  |
|  |  | **14.10. Check the mother’s record for other conditions/factors or ask her if she had:** | | | | |  |  |
|  |  | 14.10.a | | | Eclampsia |  |  |  |
|  |  | 14.10.b | | | Breech delivery |  |  |  |
|  |  | 14.10.c | | | Delivery by vacuum extraction |  |  |  |
|  |  | 14.11 | | | Ensures that the breastfeeding is initiated |  |  |  |
|  |  | 14.12 | | | Observe the newborn at the breast, if s/he is ready to feed |  |  |  |
|  |  | 14.13 | | | Ask mother to put the baby to breast to observer attachment, sucking and positioning. |  |  |  |
|  |  | **14.14 Physical Examination ( Look/Feel)** | | | | |  |  |
|  |  | 14.14.a | | | General appearance (alert or lethargic , cyanosed) |  |  |  |
|  |  | 14.14.b | | | Cry (normal , irritable or high – pitched) |  |  |  |
|  |  | 14.14.c | | | Breathing rate (normal range 30-40 |  |  |  |
|  |  | 14.14.d | | | breaths/minute),grunting , chest indrawing |  |  |  |
|  |  | 14.14.e | | | Heart rate(normal range 120-160/Minute) |  |  |  |
|  |  | 14.14.f | | | Temperature (normal range 36.5-37.5) |  |  |  |
|  |  | 14.15 | | | Weight the newborn. |  |  |  |
|  |  | 14.16 | | | Measure the head circumference |  |  |  |
|  |  | 14.17 | | | Check the skull contours and feel for the normal sutures and fontanelles |  |  |  |
|  |  | 14.18 | | | Open the eyelids and check that eyes have a normal appearance and there are no signs of infection |  |  |  |
|  |  | 14.19 | | | Check for any abnormalities of the face , especially for asymmetrical movement |  |  |  |
|  |  | 14.20 | | | Examine the upper limbs and lower limbs: Check the skin , soft tissues and bones for abnormalities |  |  |  |
|  |  | 14.21 | | | Examine the chest for symmetrical movement. |  |  |  |
|  |  | 14.22 | | | Examine the umbilicus for bleeding and check that the tie is tightly applied. |  |  |  |
|  |  | 14.23 | | | Examine the genitalia for abnormalities |  |  |  |
|  |  | 14.24 | | | Check that the anus is patent. |  |  |  |
|  |  | 14.25 | | | Examine the spine for abnormalities. |  |  |  |
|  |  | 14.26 | | | Provide counseling about danger signs in the newborn period and what to do about them |  |  |  |
|  |  | 14.27 | | | Ensures that the baby is not bathed within 3 days of birth. |  |  |  |
|  |  | 14.28 | | | Wash hands thoroughly with soap and water and dry with a clean, dry cloth or air dry. |  |  |  |
|  |  | 14.29 | | | Inform the mother of your findings and ask her if she has additional questions. |  |  |  |
|  |  | 14.30 | | | Record all relevant findings from the physical examination. |  |  |  |
|  | | **Achieved: Yes/No (Circle the answer)** | | | | |  |  |
|  | 15. The provider properly performs resuscitation of the newborn. (If need resuscitation of the newborn then 17.1 observed the following otherwise skip this section) | | 15.1 In the event of resuscitation with bag and mask | | | | |  |
|  |  |  | 15.1.a | | Places the mask so it covers the baby’s chin, mouth, and nose |  |  |  |
|  |  |  | 15.1.b | | Ensures that an appropriate seal has been formed between mask, nose, mouth and chin |  |  |  |
|  |  |  | 15.1.c | | Ventilates 40 times per minute for 1 minute |  |  |  |
|  |  |  | 15.1.d | | Pauses and determines whether the baby is breathing spontaneously  (If the baby is breathing and there is no sign of respiratory difficulty (intercostal retractions or grunting), place the baby in skin-to-skin contact with mother) |  |  |  |
|  |  |  | **15.2 If the baby does not begin to breathe or if breathing is less than 20/min minute or gasping:** | | | | |  |
|  |  |  | 15.2.a | | Continue artificial ventilation |  |  |  |
|  |  |  | 15.2.b | | Assesses the need for special care |  |  |  |
|  |  |  | 15.2.c | | Explains to the mother what is happening, if possible |  |  |  |
|  |  |  | **15.3 If there is no breathing after 20 minutes of ventilation or gasping (type of breathing )for 30 minutes** | | | | |  |
|  |  |  | 15.3.a | | Suspends resuscitation |  |  |  |
|  |  |  | 15.3.b | | Records the time of death |  |  |  |
|  |  |  | 15.4 | | Provides emotional support to mother/parents and family members |  |  |  |
|  |  |  | 15.5 | | Record all actions taken on the woman’s clinical record |  |  |  |
|  |  |  | 15.6 | | Asks the mother whether she has any questions, and responds using easy-to-understand language |  |  |  |
|  |  |  | 15.7 | | Thank the mother for coming and tell her when she should come for her next postpartum visit, if necessary |  |  |  |
|  |  | **Achieved: Yes/No (Circle the answer)** | | | | |  | |

|  | Total Score | Observed score | Achievement score | Proportion |
| --- | --- | --- | --- | --- |
| 1. Standard / Components | 15 |  |  |  |
| 2. Activities | 115 |  |  |  |

**18. Procedure done by**

| **a. Designation of the provider** | **b. which part of the procedure done** |
| --- | --- |
| **1.** | **1.** |
| **2.** | **2.** |
| **3.** | **3.** |
| **4.** | **4.** |
| **5.** | **5.** |
| **6.** | **6.** |

**Code list for designation of the provider:** 01=Consultant/Specialist in Ob/Gyn, 02=MO/Assistant Register, 03=Consultant/Specialist in Anaesthesia, 04=Consultant/Specialist in Paediatrics, 05=SSN/SN, 06=FWV/Senior FWV, 07=HA/SACMO/ MA/ Paramedics, 08= FWA, 09= CHCP/CSBA/ Community volunteer, 10=Assistant Nurse/ Student nurse , 11= ANA/Nurse AID/FMA/ Aya/ Dai nurse/ OT boy, 12= MT, 13=Sweeper/Cleaner/MLSS/Ward boy/Driver,

14= Others (specify_________________________________________________)

1. **Particulars of the primary provider:**

| 1. Sex Male = 1, Female = 2 |  | 4. Years of service | ­­­­Yrs |
| --- | --- | --- | --- |
| 2. Designation |  | 5. Years of service in this facility | Yrs |
| 3. Professional qualification/ Training | a. | b. | c. |

**Code list for Qualification:** 01=FCPS/MCPS/DGO, 02=MBBS, 03=Post graduate training, 04= EOC training, 05=Basic training (FWV/SACMO/Paramedics), 06= Basic training (CHCP/HA), 07=Diploma /BSC in nursing, 08=Midwifery, 09=SBA/TBA/CSBA training, 10=Any other short training, 11=Study in nursing, 12= Others (specify________________________________________________________________________)

1. **Particulars of the Mother:** Collect information from the health care provider at the end of the observation

| 1. Age | Yrs | 2. Para (+Abortus/miscarriage) |  |
| --- | --- | --- | --- |
| 3. Gravida |  | 4. Gestational age | Weeks |
| 5. First pregnancy  Yes = 1 , No = 2 |  | 6. Multiple Pregnancy Yes = 1 , No = 2 |  |
| 7. Type of delivery NVD=1, CS=2, Miscarraige =3, Others ____________________________________________________=4 | | | |
| 8. Any high risk indicator | a. | b. | c. |

**(Gravida**indicates the number of times the mother has been pregnant, regardless of whether these pregnancies were carried to term. A current pregnancy, if any, is included in this count. **Para** indicates the number of >20 wks births (including viable and non-viable i.e. stillbirths). Pregnancies consisting of multiples, such as twins or triplets, count as ONE birth for the purpose of this notation. **Abortus**is the number of pregnancies that were lost for any reason, including induced abortions or miscarriages. The abortus term is sometimes dropped when no pregnancies have been lost. Stillbirths are not included.)

**Code list for High risk factor:** 01=Previous C/S, 02=Pre-eclampsia /Eclampsia, 03=Bad obstetric history, 04= Malpresentation, 05=Sub-fertility, 06=Oligo-hydramnios, 07= Post dated , 08=Incomplete abortion, 09=Fetal distress, 10=Obstructed labor,11= PROM/ Leaking membrane,12= Multiple pregnancy,13=Home trialed, 14=Other Medical problem,15=PV bleeding,16= others (specify_______________________)

| **Comments** |
| --- |
|  |

**Observation End Time: |___||___|:|___||___|**

Signature of the Observer: __________________________ **Date:** ___/___/2014

Signature of the Supervisor: __________________________ **Date:** ___/___/2014

Signature of the Data entry personnel: ________________________ **Date:** ___/___/2014
