# Supplementary material for: Quality of maternal and newborn healthcare services in two public hospitals of Bangladesh: identifying gaps and provisions for improvement
Source: BMC Pregnancy Childbirth. 2019 Dec 10;19:488. doi: 10.1186/s12884-019-2656-1 (PMC6905111; doi:10.1186/s12884-019-2656-1)
Supplement: Supplementary file 5 — Additional file 5. Checklist_Infection Orevention.doc (Infection prevention checklist). [file 12884_2019_2656_MOESM5_ESM.doc]

**Appendix V: Checklists to observe quality of care of MNH Cases attending the Health Facility**

**International Centre for Diarrhoeal Disease Research, Bangladesh (icddr,b)**

**Protocol Number: PR-13098**

**Protocol Title: Operations research to improve 24/7 delivery and EmONC services in public facilities by health systems strengthening in Bangladesh.**

**AREA 9: Infection Prevention**

**Facility Name: _____________________________________ Facility Type: ______________________________**

**District: _________________________________ Upazilla: _____________________**

**UFI of the facility:** |___|___|___|___|___|___|___|___|

**Place of observation: ____________________________________**

**Code list:** 01= OPD/EPI room, 02=Ward/Cabin, 03=ANC room, 04=Labor/Delivery room, 05=OT,

06=Nurse/ SACMO/CHCP Room, 07= Others (specify_______________________________)

**ASSESSMENT TYPE:** (BASELINE 🞎/PERIODIC¨)

**Phase of Data collection:** Phase I 🞎/Phase II 🞎/Phase III 🞎

**Name of the Observer** ___________________________________

**Case no:** |___|___| **Patient no:** |___|___|___|___|

**Date:** ___/___/2014  **Observation Start Time: |___||___|:|___||___|**

**Operational definition:**

- **Done**: Performs the step or task according to the standard procedure or guidelines.
- **Not done:** Unable to perform the step or task according to the standard procedure or guidelines.
- **Not applicable**: Step or task not applicable for that particular patient during evaluation by observer.

| **PERFORMANCE STANDARDS** | **SL #** | **VERIFICATION CRITERIA** | **Observation [Done=1, Not done=0, Not applicable** | **COMMENTS** |
| --- | --- | --- | --- | --- |
| **Cleaning** | | | | |
| 1. The health facility is clean and has available clean running water. (Verify the absence of dust, blood, trash and cobwebs in the following area) | 1.1 | External area (facility compound, gardens) |  |  |
|  | 1.2 | Administrative offices |  |  |
|  | 1.3 | Admissions/reception/waiting area |  |  |
|  | 1.4 | Labour and postpartum room |  |  |
|  | 1.5 | General examination room |  |  |
|  | 1.6 | Central supply and sterilization area |  |  |
|  | 1.7 | Laboratory, Pharmacy |  |  |
|  | 1.8 | Storeroom |  |  |
|  | 1.9 | Water reservoir is well-covered and insects and animals are not able to contaminate it |  |  |
|  | 1.10 | Running water is generated from deep well, tube well |  |  |
|  | 1.11 | Water is regularly chlorinated |  |  |
|  | 1.12 | Water is tested for contamination/Arsenic regularly (once a year) |  |  |
|  | 1.13 | Water is available at all points of patient contact (in exam rooms, labor and delivery rooms, lab, vaccination room) |  |  |
|  | 1.14 | Water is available for toilets and for cleaning |  |  |
|  | Achieved: Yes = 1, No = 2 | | |  |
| 1. The facility uses an appropriate process to clean rooms in the **maternity ward,** **patient wards and clinical areas.** | **2.1 Housekeeping personnel wear personal protective equipment during cleaning:** | | |  |
|  | 2.1 a | Utility gloves |  |  |
|  | 2.1b | Plastic apron |  |  |
|  | 2.1c | Enclosed shoes |  |  |
|  | 2.2 | All waste is collected and removed from the room in closed, leak-proof containers |  |  |
|  | 2.3 | Containers with 0.5% chlorine solution with instruments are removed from the room |  |  |
|  | 2.4 | Blood and body fluid spills are mopped with 0.5% chlorine solution, and then area is cleaned with detergent and water |  |  |
|  | 2.5 | All horizontal surfaces that have come in immediate contact with a patient or body fluids are cleaned with a disinfectant cleaning solution |  |  |
|  | 2.6 | The delivery bed is cleaned, and all surfaces and mattress pads are wiped with a disinfectant-soaked, lint-free cloth |  |  |
|  | 2.7 | Instrument trolleys, baby scales and resuscitation equipment are decontaminated with a cloth dampened with 0.5% chlorine solution and rinsed with clean water |  |  |
|  | **2.8 Three buckets are use:** | | |  |
|  | 2.8.a | One with the disinfectant cleaning solution/Chlorine |  |  |
|  | 2.8.b | One with detergent ( soapy water) |  |  |
|  | 2.8.c | One with clean water for rinsing |  |  |
|  | 2.9 | Tables, chairs, shelves are wiped with soapy water daily |  |  |
|  | 2.10 | Examination bed, procedure tables, patient beds are wiped with a disinfectant cleaning solution after each patient |  |  |
|  | 2.11 | Walls, windows, doors are wiped with soapy water routinely weekly; spot cleaned when visibly dirty |  |  |
|  | 2.12 | Floors/hallways are damp mopped at least daily with a disinfectant cleaning solution |  |  |
|  | 2.13 | Trash is picked up around beds and desks and trash containers emptied frequently |  |  |
|  | 2.14 | Sinks are scrubbed daily and as necessary with separate mop, cloth or brush with a disinfectant cleaning solution |  |  |
|  | 2.15 | Puncture-resistant containers are closed and removed when three quarters full |  |  |
|  | 2.16 | Medical equipment e.g., stethoscopes, BP machines are wiped daily or are cleaned with disinfectant after each contact with infected skin |  |  |
|  | 2.17 | Thermometers are disinfected and cleaned after each contact |  |  |
|  | 2.18 | Bathrooms are cleaned daily and as necessary with separate mop, cloth or brush with a disinfectant cleaning solution |  |  |
|  | 2.19 | Hand hygiene is performed with soap and water or using a alcohol based solution after removing gloves |  |  |
|  | Achieved: Yes = 1, No = 2 | | |  |
| 1. The equipment is properly decontaminated, cleaned and dried before reuse or storage.   (for **Labour** room) | 3.1 | 0.5% chlorine solution is prepared as disinfectant cleaning solution |  |  |
|  | **3.2 The mops, buckets, brushes and cleaning cloths are:** | | |  |
|  | 3.2.a | Decontaminated by soaking for 10 minutes in 0.5% chlorine solution or other approved disinfectant |  |  |
|  | 3.2.b | Washed in detergent and water and rinsed in clean water |  |  |
|  | 3.2.c | Dried completely before reuse or storage |  |  |
|  | 3.2.d | Keep the cleaning materials in proper place |  |  |
|  | Achieved: Yes = 1, No = 2 | | |  |
| 1. The concentration and use of antiseptics (for skin and/or mucous membrane preparation) are correct.   (for **Labour** room) | 4.1 | The antiseptic concentration is labeled:   - Ethyl, isopropyl alcohol or “methylated spirit” (60%–90%), **or** - Cetrimide and chlorexidine gluconate (2%–4%) (e.g., Savlon®), **or**   Iodine preparations (1%–3%) (e.g., Lugol’s) |  |  |
|  | 4.2 | Antiseptics are prepared in small, reusable containers for daily use as appropriate |  |  |
|  | 4.3 | Reusable containers are labeled with date each time they are refilled |  |  |
|  | 4.4 | The reusable containers are thoroughly washed with soap and water, rinsed with clean water and dried before refilling |  |  |
|  | 4.5 | Gauze or cotton wool stored dry in containers without antiseptics |  |  |
|  | 4.6 | Instruments and other items are stored dry in containers without antiseptics |  |  |
|  | 4.7 | Pick-up forceps are stored dry in containers without antiseptics |  |  |
|  | Achieved: Yes = 1, No = 2 | | |  |
| 1. There is a proper area for instrument cleaning with proper traffic flow to avoid cross-contamination.   (for **Labour** room) | 5.1 | The area is well-ventilated and illuminated |  |  |
|  | 5.2 | Area for cleaning instruments is separated from the procedure areas |  |  |
|  | 5.3 | Dirty and clean items do not have contact: clean items are on one side of the room, dirty items on the other |  |  |
|  | 5.4 | There is a receiving counter for dirty items |  |  |
|  | 5.5 | There is at least one deep sink with running water for washing instruments |  |  |
|  | 5.6 | There is a counter for instruments to dry |  |  |
|  | 5.7 | There is shelf for storing clean items |  |  |
|  | 5.8 | Contaminated materials such as lines or medical waste are kept out of this room |  |  |
|  | 5.9 | Electric items are away the water area |  |  |
|  | Achieved: Yes = 1, No = 2 | | |  |
| 1. The decontamination of instruments and other articles (immediately after used and before cleaning) is performed properly.   (for **Labour** room) | 6.1 | The concentration of chlorine solution is 0.5% - **Powder chlorine**: Calcium hypochlorite (35%), 14 grams bleach powder for 1 liter water |  |  |
|  | 6.2 | A new chlorine solution is prepared at the beginning of each day or sooner if needed |  |  |
|  | 6.3 | Instruments and other items are soaked in the 0.5% chlorine solution for 10 minutes |  |  |
|  | 6.4 | Clean containers with clean 0.5% chlorine solution are used for each surgical procedure, and changed after it |  |  |
|  | 6.5 | After 10 minutes (and approximately no longer than 30 minutes), instruments and other items are removed from the chlorine solution and rinsed with clean water or cleaned immediately |  |  |
|  | Achieved: Yes = 1, No = 2 | | |  |
| 1. The process of cleaning instruments and other items is performed properly.   (for **Labour** room) | 7.1 | Wears: Utility gloves, plastic apron, gumboots or enclosed shoes, mask and eyewear protection or face shield when dealing with blood or body fluids |  |  |
|  | 7.2 | Utilizes: Soft brush, detergent (liquid or powder) |  |  |
|  | 7.3 | Scrubs instruments and other items under the surface of water, completely removing all blood and other foreign matter |  |  |
|  | 7.4 | Disassembles instruments and other items with multiple parts, and cleans in the grooves, teeth and joints with a brush |  |  |
|  | 7.5 | Rinses the instruments and other items thoroughly with clean water |  |  |
|  | 7.6 | Allows instruments and other items to air dry, or dries with a clean towel |  |  |
|  | 7.7 | After removing gloves and other personal protective equipment-washes hands with running water and soap for 10–15 seconds or rubs hands with 3–5 ml of an alcohol-based solution and dries with an individual clean towel, paper towel, or allows hands to air dry |  |  |
|  | Achieved: Yes = 1, No = 2 | | |  |
| 1. The equipment is properly decontaminated, cleaned and dried before reuse or storage.   (for **OT**) | 8.1 | 0.5% chlorine solution is prepared as disinfectant cleaning solution |  |  |
|  | **8.2 The mops, buckets, brushes and cleaning cloths are:** | | |  |
|  | 8.2.a | Decontaminated by soaking for 10 minutes in 0.5% chlorine solution or other approved disinfectant |  |  |
|  | 8.2.b | Washed in detergent and water and rinsed in clean water |  |  |
|  | 8.2.c | Dried completely before reuse or storage |  |  |
|  | 8.2.d | Keep the cleaning materials in proper place |  |  |
|  | Achieved: Yes = 1, No = 2 | | |  |
| 1. The concentration and use of antiseptics (for skin and/or mucous membrane preparation) are correct.   (for **OT**) | 9.1 | The antiseptic concentration is labeled:   - Ethyl, isopropyl alcohol or “methylated spirit” (60%–90%), **or** - Cetrimide and chlorexidine gluconate (2%–4%) (e.g., Savlon®), **or**   Iodine preparations (1%–3%) (e.g., Lugol’s) |  |  |
|  | 9.2 | Antiseptics are prepared in small, reusable containers for daily use as appropriate |  |  |
|  | 9.3 | Reusable containers are labeled with date each time they are refilled |  |  |
|  | 9.4 | The reusable containers are thoroughly washed with soap and water, rinsed with clean water and dried before refilling |  |  |
|  | 9.5 | Gauze or cotton wool stored dry in containers without antiseptics |  |  |
|  | 9.6 | Instruments and other items are stored dry in containers without antiseptics |  |  |
|  | 9.7 | Pick-up forceps are stored dry in containers without antiseptics |  |  |
|  | Achieved: Yes = 1, No = 2 | | |  |
| 1. There is a proper area for instrument cleaning with proper traffic flow to avoid cross-contamination.   (for **OT** room) | 10.1 | The area is well-ventilated and illuminated |  |  |
|  | 10.2 | Area for cleaning instruments is separated from the procedure areas |  |  |
|  | 10.3 | Dirty and clean items do not have contact: clean items are on one side of the room, dirty items on the other |  |  |
|  | 10.4 | There is a receiving counter for dirty items |  |  |
|  | 10.5 | There is at least one deep sink with running water for washing instruments |  |  |
|  | 10.6 | There is a counter for instruments to dry |  |  |
|  | 10.7 | There is shelf for storing clean items |  |  |
|  | 10.8 | Contaminated materials such as lines or medical waste are kept out of this room |  |  |
|  | 10.9 | Electric items are away the water area |  |  |
|  | Achieved: Yes = 1, No = 2 | | |  |
| 1. The decontamination of instruments and other articles (immediately after used and before cleaning) is performed properly.   (for **OT** room) | 11.1 | The concentration of chlorine solution is 0.5% - **Powder chlorine**: Calcium hypochlorite (35%), 14 grams bleach powder for 1 liter water |  |  |
|  | 11.2 | A new chlorine solution is prepared at the beginning of each day or sooner if needed |  |  |
|  | 11.3 | Instruments and other items are soaked in the 0.5% chlorine solution for 10 minutes |  |  |
|  | 11.4 | Clean containers with clean 0.5% chlorine solution are used for each surgical procedure, and changed after it |  |  |
|  | 11.5 | After 10 minutes (and approximately no longer than 30 minutes), instruments and other items are removed from the chlorine solution and rinsed with clean water or cleaned immediately |  |  |
|  | Achieved: Yes = 1, No = 2 | | |  |
| 1. The process of cleaning instruments and other items is performed properly.   (for **OT** room) | 12.1 | Wears: Utility gloves, plastic apron, gumboots or enclosed shoes, mask and eyewear protection or face shield when dealing with blood or body fluids |  |  |
|  | 12.2 | Utilizes: Soft brush, detergent (liquid or powder) |  |  |
|  | 12.3 | Scrubs instruments and other items under the surface of water, completely removing all blood and other foreign matter |  |  |
|  | 12.4 | Disassembles instruments and other items with multiple parts, and cleans in the grooves, teeth and joints with a brush |  |  |
|  | 12.5 | Rinses the instruments and other items thoroughly with clean water |  |  |
|  | 12.6 | Allows instruments and other items to air dry, or dries with a clean towel |  |  |
|  | 12.7 | After removing gloves and other personal protective equipment-washes hands with running water and soap for 10–15 seconds or rubs hands with 3–5 ml of an alcohol-based solution and dries with an individual clean towel, paper towel, or allows hands to air dry |  |  |
|  | Achieved: Yes = 1, No = 2 | | |  |
| 1. The area for wrapping and packing instruments is adequate. | 13.1 | The area is well-ventilated and illuminated |  |  |
|  | 13.2 | There is restricted access to this area |  |  |
|  | 13.3 | There is a clean work area (separated from the cleaning area) for wrapping/packing instruments with: A large work table and shelves for holding clean packages |  |  |
|  | Achieved: Yes = 1, No = 2 | | |  |
| 1. The process of packaging instruments and other items to be sterilized is performed properly. | 14.1 | The instruments are clean and dry |  |  |
|  | **14.2 Items are properly packed to be sterilized through steam sterilization (autoclave)** | | |  |
|  | 14.2a | Cloth items have been laundered, dried and are intact (have **no** holes) |  |  |
|  | 14.2b | All jointed instruments **are opened or in unlocked position** |  |  |
|  | 14.2c | All instruments are disassembled |  |  |
|  | Achieved: Yes = 1, No = 2 | | |  |
| 1. The sterilization process is performed properly according to the type of sterilizer. | **15.1 The process of loading the autoclave and/or the dry-heat sterilization is performed properly** | | |  |
|  | 15.1.a | If the autoclave is vertical, it has one or two baskets |  |  |
|  | 15.1.b | There is at least 7–8 cm of space between the packages and the walls |  |  |
|  | 15.1.c | Packs (linen, gloves) rest on their edge, in loose contact with each other |  |  |
|  | 15.1.d | Bottles, solid metal, and glass containers with dry materials are placed on their sides with lids held loosely in place |  |  |
|  | 15.1.e | Canisters, utensils, and treatment trays (if a solid tray) are on their sides |  |  |
|  | 15.1.f | Instrument trays (mesh or perforated bottom only) are placed flat on shelves or baskets |  |  |
|  | 15.1.g | Packs are within the size limit: maximum dimensions: 30 x 30 x 50 cm or 5 kg |  |  |
|  | 15.1.h | There is space between packs and containers |  |  |
|  | 15.1.i | Solutions are sterilized by themselves |  |  |
|  | 15.1.j | Gloves are sterilized by themselves and are placed in the upper shelves or baskets |  |  |
|  | 15.2 | If using steam sterilization (autoclave) 20 minutes for unwrapped items or 30 minutes for wrapped items at 121 ºC (250 ºF) in a gravity-displacement sterilizer |  |  |
|  | **15.3 The process of unloading the sterilizer (autoclave and/or the dry-heat) is performed properly to avoid contamination of sterile items -** | | |  |
|  | 15.3.a | The door is open 12–14 cm (5–6 inches) after the sterilizing cycle has been completed, and the chamber pressure gauge reaches “0” |  |  |
|  | 15.3.b | 20-30 minutes are allowed before unloading the sterilizer, for pack and instruments to dry (not applicable if drying cycle is used) |  |  |
|  | 15.3.c | Packages are dry before unloading |  |  |
|  | 15.3.d | If a loading cart is used, the cart is removed from the sterilizer and placed aside until it is cool |  |  |
|  | 15.3.e | If the packs are loaded in baskets or are placed directly in the sterilizer, baskets and/or packs are laid out on a clean surface padded with clean paper or fabric, until they are cool |  |  |
|  | 15.3.f | Unnecessary handling of the packs is avoided |  |  |
|  | 15.3.g | When packs have cooled to room temperature, they are dispensed or placed in a sterile storage area |  |  |
|  | Achieved: Yes = 1, No = 2 | | |  |
| 1. The HLD process is performed properly according to method used. | 16.1 | All cleaned, disassembled instruments are totally immersed in water before lid is closed |  |  |
|  | 16.2 | The lid is closed |  |  |
|  | 16.3 | Instruments are boiled for 20 minutes starting from the time a rolling boil begins |  |  |
|  | 16.4 | After 30 minutes, instruments are removed with HLD or sterile forceps or gloves, dried and stored in HLD containers |  |  |
|  | Achieved: Yes = 1, No = 2 | | |  |
| 1. Antiseptics, disinfectants and other supplies are available in amounts sufficient for three months of operation. | 17.1 | **Antiseptics:** Chlorhexidine gluconate (2%–4%) (e.g., Savlon®), **or**  Iodine preparations (1%–3%) (e.g., Lugol’s), **or** Pyodine |  |  |
|  | 17.2 | **Disinfectants:** Chlorine sol/powder |  |  |
|  | **17.3 Supplies:** | | |  |
|  | 17.3.a | Detergents that do not contain acid, ammonia, or ammonium |  |  |
|  | 17.3.b | Cotton wool for injections |  |  |
|  | 17.3.c | Gauzes for sterilizing |  |  |
|  | 17.3.d | Mops, buckets and cleaning cloths |  |  |
|  | 17.3.e | Exam gloves & Sterile gloves |  |  |
|  | 17.3.f | Utility gloves for cleaning personnel |  |  |
|  | 17.3.g | Eye protectors & Masks |  |  |
|  | 17.3.h | Glycerin & Hand soap |  |  |
|  | 17.3.i | Plastic buckets different colors |  |  |
|  | 17.3.j | Ceramic/ Steal basin |  |  |
|  | 17.3.k | Plastic bags |  |  |
|  | 17.3.l | Sharp box |  |  |
|  | Achieved: Yes = 1, No = 2 | | |  |
| 1. The collection of soiled linen is performed properly to avoid injuries and contamination. | 18.1 | Soiled linen is collected in leak-proof containers without being pre-soaked or washed in the ward |  |  |
|  | 18.2 | Workers wear utility gloves when collecting soiled linen |  |  |
|  | 18.3 | Linen is sent to the laundry in closed containers (buckets, plastic bags or carts) for sorting, washing, and drying |  |  |
|  | Achieved: Yes = 1, No = 2 | | |  |
| 1. General Biosafety and IP practices are followed in the laboratory. | 19.1 | Staff is forbidden to eat, drink or smoke in the laboratory |  |  |
|  | 19.2 | It is forbidden to store food and beverages in refrigerators used for clinical specimens |  |  |
|  | 19.3 | Specimen containers have lids |  |  |
|  | 19.4 | Laboratory workers wear exam gloves to receive the specimens |  |  |
|  | 19.5 | Specimens are placed in a container that prevents leakage during collection, handling, processing, storage or transportation |  |  |
|  | 19.6 | Staff change gloves and wash hands between patients and procedures |  |  |
|  | 19.7 | Staff record information, answer the phone, open doors without gloves |  |  |
|  | Achieved: Yes = 1, No = 2 | | |  |

|  | Total Score | Observed score | Achievement score | Proportion |
| --- | --- | --- | --- | --- |
| 1. Standard / Components | 19 |  |  |  |
| 2. Activities | 156 |  |  |  |

**20.** Procedure done by

| **a. Designation of the provider** | **b. which part of the procedure done** |
| --- | --- |
| **1.** | **1.** |
| **2.** | **2.** |
| **3.** | **3.** |
| **4.** | **4.** |
| **5.** | **5.** |
| **6.** | **6.** |

**Code list for designation of the provider:** 01=Consultant/Specialist in Ob/Gyn, 02=MO/Assistant Register, 03=Consultant/Specialist in Anaesthesia, 04=Consultant/Specialist in Paediatrics, 05=SSN/SN, 06=FWV/Senior FWV, 07=HA/SACMO/ MA/ Paramedics, 08= FWA, 09= CHCP/CSBA/ Community volunteer, 10=Assistant Nurse/ Student nurse , 11= ANA/Nurse AID/FMA/ Aya/ Dai nurse/ OT boy, 12= MT, 13=Sweeper/Cleaner/MLSS/Ward boy/Driver,

14= Others (specify_________________________________________________)

| **Comments** |
| --- |
|  |

**Observation End Time: |___||___|:|___||___|**

Signature of the Observer: __________________________ **Date:** ___/___/2014

Signature of the Supervisor: __________________________ **Date:** ___/___/2014

Signature of the Data entry personnel: ________________________ **Date:** ___/___/2014
