# Supplementary material for: Quality of maternal and newborn healthcare services in two public hospitals of Bangladesh: identifying gaps and provisions for improvement
Source: BMC Pregnancy Childbirth. 2019 Dec 10;19:488. doi: 10.1186/s12884-019-2656-1 (PMC6905111; doi:10.1186/s12884-019-2656-1)
Supplement: Supplementary file 6 — Additional file 6. Checklist_Management of Maternal Complications.docx (Maternal complications management checklist). [file 12884_2019_2656_MOESM6_ESM.docx]

**Appendix V: Checklists to observe quality of care of MNH Cases attending the Health Facility**

**International Centre for Diarrhoeal Disease Research, Bangladesh (icddr,b)**

**AREA 7: MANAGEMENT OF ANTENATAL, INTRAPARTUM AND POSTPARTUM COMPLICATIONS**

**Facility Name: _____________________________________ Facility Type: ______________________________**

**District: _________________________________ Upazilla: _____________________**

**UFI of the facility:** |___|___|___|___|___|___|___|___|

**Place of observation: ____________________________________**

**Code list:** 01= OPD/EPI room, 02=Ward/Cabin, 03=ANC room, 04=Labor/Delivery room, 05=OT,

06=Nurse/ SACMO/CHCP Room, 07= Others (specify_______________________________)

**ASSESSMENT TYPE:** (BASELINE 🞎/PERIODIC🞎)

**Phase of Data collection:** Phase I 🞎/Phase II 🞎/Phase III 🞎

**Name of the Observer** ___________________________________

**Case no:** |___|___| **Patient no:** |___|___|___|___|

**Date:** ___/___/2014  **Observation Start Time: |___||___|:|___||___|**

**Operational definition:**

- **Done**: Performs the step or task according to the standard procedure or guidelines.
- **Not done:** Unable to perform the step or task according to the standard procedure or guidelines.
- **Not applicable**: Step or task not applicable for that particular patient during evaluation by observer.

| **PERFORMANCE**  **STANDARDS** |  | **VERIFICATION CRITERIA** | **Observation**  **[Done=1, Not done=0,**  **Not applicable=9** | **COMMENTS** |
| --- | --- | --- | --- | --- |
| 1. The health facility has readily available and in working conditions the basic equipment, supplies and medicine for managing obstetric complications. (**Ask the provider at the emergency room to show the following)** | **1.1 Equipment and supplies:** | | |  |
|  | 1.1.a | Sphygmomanometer**,** thermometer, container for taking blood sample |  |  |
|  | 1.1.b | Gloves, 16- or 18-gauge needle or cannula, IV cut down set, IV equipment, syringes |  |  |
|  | 1.1.c | Ambu bag for adult |  |  |
|  | 1.1.d | Oxygen tank or central supply source, cannula, and mask |  |  |
|  | 1.1.e | Bladder catheter and urine collection bags |  |  |
|  | 1.1.f | Rubber hammer for assessing reflexes |  |  |
|  | 1.1.g | Dipsticks test for protein, Clean lab tubes |  |  |
|  | 1.2 Medicine for managing obstetric complication available at the emergency room: | | |  |
|  | 1.2.a | Saline or Ringer’s lactate solution |  |  |
|  | 1.2.b | Oxytocin and ergometrine |  |  |
|  | 1.2.c | Magnesium sulphate 20% and 50% solution |  |  |
|  | 1.2.d | Diazepam 10 mg |  |  |
|  | 1.2.e | Lignocaine 2% |  |  |
|  | 1.2.f | Calcium gluconate 10% solution |  |  |
|  | 1.2.g | Hydralazine or Nifedipine, Antibiotics: Ampicillin, Penicillin, Metronidazole, Gentamicin |  |  |
|  | **Achieved: Yes/No (Circle the answer)** | | |  |
| 2. The provider describes the management of shock. (Hypovolemic shock). **Instructions to the assessor:** Assess the management of hypovolemic shock by interviewing one provider who works in the maternity or in the emergency room using the tool.) | **2.1 What is the immediate treatment to be given?** | | |  |
|  | 2.1.a | Maintains airways |  |  |
|  | 2.1.b | Administers oxygen to the women, 6-8 L/minute by cannula or mask |  |  |
|  | 2.1.c | Starts two IV lines using a 16-or 18- gauge needle |  |  |
|  | 2.1.d | Performs IV cut down if the vein is not accessible |  |  |
|  | 2.1.e | Takes a blood sample for performing haemoglobin, coagulation and blood group and Rh (cross- matching) |  |  |
|  | 2.1.f | Requests blood |  |  |
|  | **2.2 Replaces fluids:** | | |  |
|  | 2.2.a | With saline or Ringer’s solution |  |  |
|  | 2.2.b | 1 Litre in a 15-20 minute period (wide open rate) in each line |  |  |
|  | 2.2.c | Labels IV bags with bed number and medications added, if any |  |  |
|  | 2.2.d | Administer at least two additional liters of this solution during the first hour |  |  |
|  | 2.2.e | Continues to replace volume IV in accordance with the loss of blood (two or three times the estimated loss) |  |  |
|  | 2.2.f | Assesses women’s need for transfusion based upon signs and symptoms of shock or impending shock due to amount of blood lost |  |  |
|  | 2.3 | Performs bladder catheterization |  |  |
|  | 2.4 | keeps the women warm |  |  |
|  | **Achieved: Yes/No (Circle the answer)** | | |  |
| 3. The provider describes how to evaluate patient’s response and next steps. | **3.1 Re-assesses the woman every 15 minutes** | | |  |
|  | 3.1.a | pulse |  |  |
|  | 3.1.b | BP |  |  |
|  | 3.1.c | Mental state (confusion) |  |  |
|  | 3.1.d | Fluid balance |  |  |
|  | 3.1.e | Keeps the woman warmly covered |  |  |
|  | 3.2 | Adjust IV infusion to 1 Litre a six-hour period (60 drops/minute) |  |  |
|  | 3.3 | Continues to monitor vital signs and loss of blood every 30 minutes |  |  |
|  | **3.4 What would you do if the conditions do not improve?** | | |  |
|  | 3.4.a | Continues IV solution 1 Litre over a six-hour period (60 drops/minute) |  |  |
|  | 3.5.b | Continues to administer oxygen 6-8 L/minute |  |  |
|  | 3.6.c | Continues to monitor vital sing and fluid balance |  |  |
|  | 3.7 | Records information related to findings and procedures performed on the clinical history |  |  |
|  | **Achieved: Yes/No (Circle the answer)** | | |  |
| **For Incomplete abortion go question no. 4**  **For pre-eclampsia/Eclampsia go question no. 6**  **For PPH go question no. 8** | | | | |
| **For Incomplete abortion** | | | |  |
| 4. There is a record with basic information that supports the diagnosis of incomplete abortion.  (Determine whether the following information is recorded) | 4.1 | Amenorrhoea or gestational age of 28 weeks or less |  |  |
|  | 4.2 | Prolonged and /or heavy vaginal bleeding |  |  |
|  | 4.3 | Lower abdominal pain and/or cramping |  |  |
|  | 4.4 | Vital signs |  |  |
|  | 4.5 | Open cervical os |  |  |
|  | 4.6 | Clear recording of the diagnosis or suspicion of incomplete abortion |  |  |
|  | 4.7 | Assessment for shock |  |  |
|  | **Achieved: Yes/No (Circle the answer)** | | |  |
| 5. There is a record of how the incomplete abortion was managed.  (Determine whether the following information is recorded) | **5.1 Monitoring of vital signs:** | | |  |
|  | 5.1.a | Temperature |  |  |
|  | 5.1.b | BP |  |  |
|  | 5.1.c | Pulse |  |  |
|  | 5.1.d | Breathing |  |  |
|  | 5.2 | Management of shock as appropriate |  |  |
|  | 5.3 | Administration of antibiotics, if infection (fever>38^0^C, tender uterus, foul- smelling vaginal discharge). |  |  |
|  | 5.4 | Evacuation of the uterus using manual vacuum aspiration (if less than 16 weeks gestation) or dilatation and curettage |  |  |
|  | 5.5 | Lab exams requested as needed |  |  |
|  | **Achieved: Yes/No (Circle the answer)** | | |  |
| **For pre-eclampsia/Eclampsia** | | | |  |
| 6. There is a record with basic information that supports the diagnosis severe pre-eclampsia and/or eclampsia. (Determine whether the following information is recorded) [Review the one most recent case of severe pre-eclampsia or eclampsia in the clinical records (no more than six months old] | **6.1 Severe pre-eclampsia :** | | |  |
|  | 6.1.a | Diastolic BP 110 mmHg or more |  |  |
|  | 6.1.b | 20 weeks or more of gestation |  |  |
|  | 6.1.c | Proteinuria 3+ |  |  |
|  | **6.2 Eclampsia :** | | |  |
|  | 6.2.a | Convulsions |  |  |
|  | 6.2.b | Diastolic BP 90 mmHg or more |  |  |
|  | 6.2.c | 20 weeks or more of gestation |  |  |
|  | 6.2.d | Proteinuria 2+ or more |  |  |
|  | **Achieved: Yes/No (Circle the answer)** | | |  |
| 7. There is a record of the management of severe pre-eclampsia and/or eclampsia | **7.1 If diastolic BP is 90 mmHg or more :** | | |  |
|  | 7.1.a | Initiate magnesium sulfate treatment immediately |  |  |
|  | 7.1.b | Start IV line with saline or Ringer’s lactate solution |  |  |
|  | **7.2 Initial dose of magnesium sulfate :** | | |  |
|  | 7.2.a | Administers 4g of 20% magnesium sulfate in solution (20 ml) IV over a 5- minute period |  |  |
|  | 7.2.b | Administers 5 g of 50% magnesium sulfate solution (20 ml) with 1 ml of 2% lidocaine IM deep in each buttock (total 10g) |  |  |
|  | 7.3. | In the event of a second convulsion after 15 minutes, administers 2 g of 20% magnesium sulfate in solution (4 ml) IV over a 5-minute period |  |  |
|  | **7.4 Simultaneously, there is record of :** | | |  |
|  | 7.4.a | Bladder catheterization |  |  |
|  | 7.4.b | Intake and output monitoring every shift |  |  |
|  | 7.4.c | Monitoring of vital signs : |  |  |
|  | 7.4.d | BP |  |  |
|  | 7.4.e | Pulse |  |  |
|  | 7.4.f | Breathing |  |  |
|  | 7.4.g | Monitoring of foetal heart rate |  |  |
|  | 7.4.h | Performance and evaluation of clotting tests |  |  |
|  | **7.5 If there were convulsions, birth took place within 12 hours following the Convulsion or, in the absence of convulsions, within 24 hours.** | |  |  |
|  | 7.5.a | Antihypertensive treatment (if diastolic BP is 110 mmHg or more): |  |  |
|  | 7.5.b | Plan 1 : Hydralazine 5 mg IV slowly every 5 minutes, repeats at 30 min interval or 12.5 mg IM every 2 hours (Do not give more than 20 mg in total), until diastolic BP stabilizes between 90 and 100 mmHg **OR** Plan 2 : Nifedipine 5 mg sublingual, repeating the dose if the diastolic BP is still more than 110 after 10 minutes |  |  |
|  | 7.5.c | After providing the immediate treatment refers the woman for further management |  |  |
| **For management of PPH** | | |  |  |
| 8. The provider properly records the basic information to support the diagnosis of PPH. | 8.1 | Increased vaginal bleeding within the first 24 hours after childbirth (immediate PPH) or increased vaginal bleeding after the first 24 hours after delivery (delayed PPH) |  |  |
|  | **Achieved: Yes/No (Circle the answer)** | | |  |
| 9. The provider properly performs the general management of PPH.( Verify by direct observation ) | **Whether the provider immediately measures the vital signs.)** | | |  |
|  | 9.1 | Pulse |  |  |
|  | 9.2 | BP |  |  |
|  | 9.3 | Respirations |  |  |
|  | 9.4 | Level of consciousness |  |  |
|  | 9.5 | If there is shock or suspicion of shock (weak, fast pulse [110 or more per minute], systolic BP less than 90 mmHg, pallor, cold and perspiring skin, rapid breathing, confusion or unconsciousness): **Calls for help** |  |  |
|  | 9.6 | Covers the woman and elevates feet higher than heart |  |  |
|  | 9.7 | Starts oxygen at 6-8 L/minute |  |  |
|  | 9.8 | Starts two IV lines using 16- or 18-gauge needle |  |  |
|  | 9.9 | Takes a blood sample for haemoglobin, cross-matching, and clotting test |  |  |
|  | 9.10 | Initiates IV infusion with saline or Ringer’s lactate |  |  |
|  | 9.11 | Infuses 1 L in each line over a 15-20 minute period (wide open rate) |  |  |
|  | 9.12 | Administers at least two additional liters of solution during the first hour |  |  |
|  | 9.13 | Continues to replace volume IV according to blood loss |  |  |
|  | 9.14 | Monitors vital signs every 15 minutes |  |  |
|  | 9.15 | Performs bladder catheterization |  |  |
|  | 9.16 | Monitors fluid intake and output every 30 minutes |  |  |
|  | 9.17 | Administers 10 IU of oxytocin IM |  |  |
|  | **Achieved: Yes/No (Circle the answer)** | | |  |
| 10. The provider properly performs the specific management of the cause of the PPH. (**Verify by direct observation, )** | 10.1 | Identifies the cause of bleeding and initiates specific management immediately |  |  |
|  | **10.2 IF bleeding due to retained placenta :** | |  |  |
|  | 10.2.a | Attempts placental extraction by applying controlled cord traction (with counter traction on uterus), asking the woman to bear down. |  |  |
|  | 10.2.b | If placenta is not expelled, administers another dose of oxytocin 10 IU IM or 20 IU in 500 ml of Ringer’s Lactate at 40-60 drops/min (do not use ergometrine) and repeats controlled cord traction |  |  |
|  | 10.2.c | If this maneuver fails, performs manual extraction of placenta |  |  |
|  | 10.2 d | Put high-level disinfected gloves on both hands (use elbow-length gloves if available), |  |  |
|  | 10.2 e | Hold the umbilical cord with a clamp, pulling the cord gently until it is parallel to the floor, |  |  |
|  | 10.2 f | Place the fingers of right hand into the vagina and the uterine cavity and follow the canal until reaches up to the insertion of the cord, |  |  |
|  | 10.2 g | When the placenta has been located, let go of the cord and move left hand on the abdomen to support the fundus of the uterus to prevent uterine perforation, |  |  |
|  | 10.2 h | Move the fingers of the hand in the uterus laterally until the edge of the placenta is located, |  |  |
|  | 10.2 i | Keeping the fingers tightly together, ease the edge of the hand gently between the placenta and the uterine wall, with the palm facing the placenta, Gradually move the hand back and forth in a smooth lateral motion until the whole placenta is separated from the uterine wall: |  |  |
|  | 10.2 j | When the placenta is completely separated:  Palpate the inside of the uterine cavity to ensure that all placental tissue has been remove, Slowly withdraw the hand from the uterus bringing the placenta with it. Continue to provide counter- traction to the funds by pushing it in the opposite direction of the hand that is being withdrawn, As the placenta is visible at the vulva cup the placenta in both hands. |  |  |
|  | 10.2.k | In the event of manual extraction of placenta, administers 500 mg 6 hourly for 7 days. single dose of ampicillin 2 g IV or penicillin 5 million IU IV PLUS metronidazole 500 mg IV (single dose) |  |  |
|  | 10.2 l | If the placenta does not separate from the uterine wall by gentle lateral movement of the fingers at the line of cleavage, suspect placenta accreta and arrange for surgical intervention. Refers for hysterectomy in case of placenta accrete |  |  |
|  | **Achieved: Yes/No (Circle the answer)** | | |  |
|  | **IF bleeding due to uterine atony :** | |  |  |
|  | **10.3 Explains to the woman what she is going to do, then** | | |  |
|  | 10.3.a | Performs vigorous uterine massage |  |  |
|  | 10.3.b | Administers oxytocin 20 IU in 1 L of saline solution, 60 drops/minute, and ergometrine 0.2 mg IM or IV (if the woman is not hypertensive) |  |  |
|  | 10.3.c | Continues to administer oxytocin 20 IU in 1 L of saline solution, 40 drops/minute, up to a maximum of 3 L of solution with oxytocin, if necessary, and ergometrine 0.2 mg IM or IV 15 minutes every four hours, up to three times if necessary (if the woman is not hypertensive). |  |  |
|  | 10.3.d | If haemorrhaging continues, performs bi-manual uterine compression |  |  |
|  | 10.3.e | If still bleeding refers the patient for further management |  |  |
|  | **If bleeding due to retained placenta or parts :** | |  |  |
|  | **IF bleeding due to perineal or cervical tears :** | |  |  |
|  | 10.4.a | Explains the problem and tells the woman what she is going to do |  |  |
|  | 10.4 b | Put high-level disinfected gloves on both hands. |  |  |
|  | 10.4 c | Cleanse the vagina and cervix with an antiseptic solution, |  |  |
|  | 10.4 d | Give anesthesia, if necessary (IV pethidine and diazepam) |  |  |
|  | 10.4 e | If necessary Take some steps to empty the bladder or insert a catheter, if necessary, |  |  |
|  | 10.4 f | Grasp the cervix on either side of the laceration using ring or sponge forceps, Place the first suture at the top of the tear and close it with a continuous suture, including the whole thickness of the cervix each time the suture needle is inserted. If a long section of the rim of the cervix is tattered, run it with a continuous suture. Use ring forceps if the apex is difficult to reach and legate. |  |  |
|  | **Achieved: Yes/No (Circle the answer)** | | |  |
| 11. The provider properly performs follow up in a PPH case.( Verify by direct observation) | 11.1 | Monitors uterine contraction, vital signs, and bleeding every 15 minutes during the first two hours. |  |  |
|  | 11.2 | Performs uterine massage and extraction of clots |  |  |
|  | 11.3 | Measures intake and output hourly |  |  |
|  | 11.4 | Replaces volume and transfuses if necessary |  |  |
|  | 11.5 | Measures hematocrit of haemoglobin 24 hours after haemorrhage has been controlled |  |  |
|  | 11.6 | If hematocrit is less than 20% or hemoglobin less than 7 g/dL, prescribes 120 mg of iron sulfate and 400 mcg of folic acid orally for a period of three months |  |  |
|  | 11.7 | Records all information in the patient’s chart |  |  |
|  | 11.8 | Explains to the mother and her husband what happened and what the intervention was |  |  |
|  | **Achieved: Yes/No (Circle the answer)** | | |  |

**For Incomplete Abortion:**

|  | Total Score | Observed score | Achievement score | Proportion |
| --- | --- | --- | --- | --- |
| 1. Standard / Components | 5 |  |  |  |
| 2. Activities | 25 |  |  |  |

**For Pre-Eclampsia**

|  | Total Score | Observed score | Achievement score | Proportion |
| --- | --- | --- | --- | --- |
| 1. Standard / Components | 4 |  |  |  |
| 2. Activities | 19 |  |  |  |

**For Eclampsia**

|  | Total Score | Observed score | Achievement score | Proportion |
| --- | --- | --- | --- | --- |
| 1. Standard / Components | 4 |  |  |  |
| 2. Activities | 19 |  |  |  |

**For Retained Placenta**

|  | Total Score | Observed score | Achievement score | Proportion |
| --- | --- | --- | --- | --- |
| 1. Standard / Components | 7 |  |  |  |
| 2. Activities | 32 |  |  |  |

**For Utrine Atony**

|  | Total Score | Observed score | Achievement score | Proportion |
| --- | --- | --- | --- | --- |
| 1. Standard / Components | 7 |  |  |  |
| 2. Activities | 32 |  |  |  |

**For Cervical/Perineal Tear**

|  | Total Score | Observed score | Achievement score | Proportion |
| --- | --- | --- | --- | --- |
| 1. Standard / Components | 7 |  |  |  |
| 2. Activities | 32 |  |  |  |

1. **Procedure done by**

| **a. Designation of the provider** | **b. which part of the procedure done** |
| --- | --- |
| **1.** | **1.** |
| **2.** | **2.** |
| **3.** | **3.** |
| **4.** | **4.** |
| **5.** | **5.** |
| **6.** | **6.** |

**Code list for designation of the provider:** 01=Consultant/Specialist in Ob/Gyn, 02=MO/Assistant Register, 03=Consultant/Specialist in Anaesthesia, 04=Consultant/Specialist in Paediatrics, 05=SSN/SN, 06=FWV/Senior FWV, 07=HA/SACMO/ MA/ Paramedics, 08= FWA, 09= CHCP/CSBA/ Community volunteer, 10=Assistant Nurse/ Student nurse , 11= ANA/Nurse AID/FMA/ Aya/ Dai nurse/ OT boy, 12= MT, 13=Sweeper/Cleaner/MLSS/Ward boy/Driver,

14= Others (specify_________________________________________________)

1. **Particulars of the primary provider:**

| 1. Sex Male = 1, Female = 2 |  | 4. Years of service | ­­­­Yrs |
| --- | --- | --- | --- |
| 2. Designation |  | 5. Years of service in this facility | Yrs |
| 3. Professional qualification/ Training | a. | b. | c. |

**Code list for Qualification:** 01=FCPS/MCPS/DGO, 02=MBBS, 03=Post graduate training, 04= EOC training, 05=Basic training (FWV/SACMO/Paramedics), 06= Basic training (CHCP/HA), 07=Diploma /BSC in nursing, 08=Midwifery, 09=SBA/TBA/CSBA training, 10=Any other short training, 11=Study in nursing, 12= Others (specify________________________________________________________________________)

1. **Particulars of the Mother:** Collect information from the health care provider at the end of the observation

| 1. Age | Yrs | 2. Para (+Abortus/miscarriage) |  |
| --- | --- | --- | --- |
| 3. Gravida |  | 4. Gestational age | Weeks |
| 5. First pregnancy Yes = 1 , No = 2 |  | 6. Multiple Pregnancy Yes = 1 , No = 2 |  |
| 7. Type of delivery NVD=1, CS=2, Miscarraige =3, Others ____________________________________________________=4 | | | |
| 8. Any high risk indicator | a. | b. | c. |

**(Gravida**indicates the number of times the mother has been pregnant, regardless of whether these pregnancies were carried to term. A current pregnancy, if any, is included in this count. **Para** indicates the number of >20 wks births (including viable and non-viable i.e. stillbirths). Pregnancies consisting of multiples, such as twins or triplets, count as ONE birth for the purpose of this notation. **Abortus**is the number of pregnancies that were lost for any reason, including induced abortions or miscarriages. The abortus term is sometimes dropped when no pregnancies have been lost. Stillbirths are not included.)

**Code list for High risk factor:** 01=Previous C/S, 02=Pre-eclampsia /Eclampsia, 03=Bad obstetric history, 04= Malpresentation, 05=Sub-fertility, 06=Oligo-hydramnios, 07= Post dated , 08=Incomplete abortion, 09=Fetal distress, 10=Obstructed labor,11= PROM/ Leaking membrane,12= Multiple pregnancy,13=Home trialed, 14=Other Medical problem,15=PV bleeding,16= others (specify_______________________)

| **Comments** |
| --- |
|  |

**Observation End Time: |___||___|:|___||___|**

Signature of the Observer: __________________________ **Date:** ___/___/2014

Signature of the Supervisor: __________________________ **Date:** ___/___/2014

Signature of the Data entry personnel: ________________________ **Date:** ___/___/2014
