# Supplementary material for: Quality of maternal and newborn healthcare services in two public hospitals of Bangladesh: identifying gaps and provisions for improvement
Source: BMC Pregnancy Childbirth. 2019 Dec 10;19:488. doi: 10.1186/s12884-019-2656-1 (PMC6905111; doi:10.1186/s12884-019-2656-1)
Supplement: Supplementary file 7 — Additional file 7. Checklist_PNC.doc (PNC checklist). [file 12884_2019_2656_MOESM7_ESM.doc]

**Appendix V: Checklists to observe quality of care of MNH Cases attending the Health Facility**

**International Centre for Diarrhoeal Disease Research, Bangladesh (icddr,b)**

**AREA 4: POSTNATAL CARE (within 42 days of delivery)**

**Facility Name: _____________________________________ Facility Type: ______________________________**

**District: _________________________________ Upazilla: _____________________**

**UFI of the facility:** |___|___|___|___|___|___|___|___|

**Place of observation: ____________________________________**

**Code list:** 01= OPD/EPI room, 02=Ward/Cabin, 03=ANC room, 04=Labor/Delivery room, 05=OT,

06=Nurse/ SACMO/CHCP Room, 07= Others (specify_______________________________)

**Assessment Type:** (BASELINE 🞎/PERIODIC¨)

**Phase of Data collection:** Phase I 🞎/Phase II 🞎/Phase III 🞎

**Name of the Observer** ___________________________________

**Case no:** |___|___| **Patient no:** |___|___|___|___|

**Date:** ___/___/2014  **Observation Start Time: |___||___|:|___||___|**

**Operational definition:**

- **Done**: Performs the step or task according to the standard procedure or guidelines.
- **Not done:** Unable to perform the step or task according to the standard procedure or guidelines.
- **Not applicable**: Step or task not applicable for that particular patient during evaluation by observer.
- Day of Postnatal period:

| **PERFORMANCE STANDARDS** |  | **VERIFICATION CRITERIA** | **Observation**  **[Done=1, Not done=0,**  **Not applicable=9** | **COMMENTS** |
| --- | --- | --- | --- | --- |
| 1. The provider does a rapid initial assessment at first contact with the woman. Observe whether the provider. | 1.1 | Greets the client and her companion. Speaks in easy to understand language |  |  |
|  | 1.2 | Did the care provider introduce himself/herself to the mother |  |  |
|  | 1.3 | Encourages her husband or companion to remain at her side (if appropriate) |  |  |
|  | **1.4 Asks the woman if she is currently experiencing any of the following:-** | | |  |
|  | 1.4.a | Heavy vaginal bleeding |  |  |
|  | 1.4.b | respiratory difficulty |  |  |
|  | 1.4.c | fever |  |  |
|  | 1.4.d | severe headache/blurred vision |  |  |
|  | 1.4.e | severe abdominal pain |  |  |
|  | 1.4.f | convulsions/loss of consciousness |  |  |
|  | 1.4.g | foul smelling vaginal discharge |  |  |
|  | 1.4.h | High blood pressure |  |  |
|  | 1.5 | Ensure the date of delivery and the day of post natal period (record review) |  |  |
|  | 1.6 | Records findings in chart |  |  |
|  | Achieved: Yes = 1, No = 2 | | |  |
| 2.The provider receives and treats the woman cordially and respectfully.( Observe whether the provider) | 2.1 | Encourages the woman to ask her husband or companion to remain at her side, as appropriate |  |  |
|  | 2.2 | Explains to the woman and her husband or companion what she/he is going to do and encourages her to ask questions |  |  |
|  | Achieved: Yes = 1, No = 2 | | |  |
| 3.The provider verifies the existence of or opens a clinical record for the woman. (Observe/determine whether the provider verifies the following) | 3.1 | The client has clinical history |  |  |
|  | 3.2 | If no clinical history exists, the provider takes the history on: |  |  |
|  | 3.3 | Personal information and social history |  |  |
|  | 3.4 | Medical history (UTI) |  |  |
|  | 3.5 | History of antenatal check-up, Obstetrical history, including details of the birth and immediate postnatal period |  |  |
|  | Achieved: Yes = 1, No = 2 | | |  |
| 4. The provider conducts a routine physical exam. (Observe whether the provider does the following) | 4.1 | Washes hands with soap and water and dries them with a clean towel or uses a alcohol based solution |  |  |
|  | 4.2 | Ensures privacy: |  |  |
|  | **4.3 Measures vital signs –** | | |  |
|  | 4.3.a | Temperatures |  |  |
|  | 4.3.b | Pulse |  |  |
|  | 4.3.c | Respiration |  |  |
|  | 4.3.d | BP |  |  |
|  | 4.4 | Checks conjunctiva for pallor and sclera for jaundice |  |  |
|  | 4.5 | Examines breasts for establishment of lactation, engorgement and tenderness and cracked/inverted nipples |  |  |
|  | 4.6 | Examines abdomen for involution of uterus, tenderness and distension |  |  |
|  | 4.7 | Asks if bladder and bowel function are normal |  |  |
|  | 4.8 | Checks lower legs for oedema, tenderness |  |  |
|  | 4.9 | With woman’s permission, reviews her perineum for inflammation, status of episiotomy/tears. |  |  |
|  | 4.10 | Reviews the pad and lochia for colour, amount, consistency and odour |  |  |
|  | 4.11 | Asks the woman about any anxiety, depression or preoccupations she may have |  |  |
|  | Achieved: Yes = 1, No = 2 | | |  |
| 1. The provider properly manages the postnatal mother according to the findings of the assessment. (Observe whether the provider counsels the mother and her family on) | 5.1 | Nutrition (animal proteins, legumes, green vegetables, fats, carbohydrates, fruits and vitamins) |  |  |
|  | 5.2 | Importance of taking regular meals containing mixed foods |  |  |
|  | 5.3 | Importance of taking enough fluids, 2–3 liters of water daily |  |  |
|  | 5.4 | Personal and environmental hygiene |  |  |
|  | 5.5 | Perineal care (bathing, changing pads/cloths) |  |  |
|  | 5.6 | Adequate rest and sleep |  |  |
|  | **5.7 Activites to be avoided;** | | |  |
|  | 5.7.a | a) heavy work |  |  |
|  | 5.7.b | b) coitus |  |  |
|  | 5.8 | Exercise for perineum and lower abdominal muscles |  |  |
|  | 5.9 | Family planning including birth spacing |  |  |
|  | 5.10 | Was the baby full term |  |  |
|  | 5.11 | Baby’s bladder/bowel function |  |  |
|  | 5.12 | Any problem or concerns about the baby now |  |  |
|  | 5.13 | BCG and OPV dose schedule |  |  |
|  | 5.14 | Exclusive breastfeeding for six months and weaning advice after six months (begin soft foods) |  |  |
|  | 5.15 | Need for other postnatal checks during the first six days after delivery and again at six weeks |  |  |
|  | 5.16 | Resumption of sexual activity after six weeks or when sees fit, including counselling client on discussing this topic with husband. |  |  |
|  | **5.17 Gives to the mother the following with explanation:** | | |  |
|  | 5.17.a | analgesia if required |  |  |
|  | 5.17.b | viitamin A |  |  |
|  | 5.17.c | Ferus Sulfat per MOH guidelines |  |  |
|  | 5.17.d | anti-malarial tablets (based on region/population-specific need), |  |  |
|  | 5.17.e | mebendazole (based on region/population-specific need) |  |  |
|  | 5.18 | Refers or notifies physician if complications develop |  |  |
|  | 5.19 | Arranges the next appointment if required |  |  |
|  | 5.20 | Records all information in the mother’s chart |  |  |
|  | Achieved: Yes = 1, No = 2 | | |  |
| 6.The provider properly advises the mother and her husband /companion on danger signs during the postpartum.( Observe whether) | **6. The provider explains to the mother and her husband or another family member the need to come to the health facility when the following danger signs are observed:** | | |  |
|  | 6.1 | excessive vaginal bleeding, |  |  |
|  | 6.2 | dizziness, |  |  |
|  | 6.3 | severe headache, |  |  |
|  | 6.4 | convulsions, |  |  |
|  | 6.5 | severe abdominal pains, |  |  |
|  | 6.6 | heavy and/or foul smelling vaginal discharge, |  |  |
|  | 6.7 | fever, |  |  |
|  | 6.8 | blurred vision, |  |  |
|  | 6.9 | bowel and urinary dysfunction |  |  |
|  | Achieved: Yes = 1, No = 2 | | |  |
| 1. The provider refers women when required.( Verify by direct observation/interview whether the provider while referring) | 7.1 | Stabilizes the woman before leaving health facility |  |  |
|  | 7.2 | Refers the women based on protocol |  |  |
|  | 7.3 | Explains to the woman/husband/her companion the reason and site of referral with referral record |  |  |
|  | 7.4 | Makes sure to receive feedback from referral site |  |  |
|  | Achieved: Yes = 1, No = 2 | | |  |
| 1. The provider properly assesses the condition of the neonate at the first visit. (Observe if the provider during one of first visits of a neonate) | 8.1 | Ensures that the note, date and time of delivery |  |  |
|  | 8.2 | Washes hands with soap and water and dries them with a clean towel or use an alcohol-based solution |  |  |
|  | 8.3 | Weighs the baby |  |  |
|  | 8.4 | Keeps the baby warm (proper wrapping) |  |  |
|  | **Assesses the neonate from head to toe as follows:** | | |  |
|  | 8.5 | general condition/appearance of the baby, |  |  |
|  | 8.6 | axiliary temperature |  |  |
|  | 8.7 | respiratory rate (count for one minute while the baby is quiet) |  |  |
|  | 8.8 | head |  |  |
|  | 8.9 | face |  |  |
|  | 8.10 | neck and eyes |  |  |
|  | 8.11 | oral cavity |  |  |
|  | 8.12 | hands and fingers |  |  |
|  | 8.13 | chest, |  |  |
|  | 8.14 | abdomen and umbilical cord, |  |  |
|  | 8.15 | external genitalia including anal opening, |  |  |
|  | 8.16 | back and spine, |  |  |
|  | 8.17 | legs and toes |  |  |
|  | 8.18 | Refers and arranges transfer to higher center after informing the mother/relatives in the event of any serious abnormality with proper referral slip |  |  |
|  | Achieved: Yes = 1, No = 2 | | |  |
| 9. The provider properly manages the neonate. (Observe if the provider) | 9.1 | Washes hands with soap and water and dries them with a clean towel or uses an alcohol-based solution |  |  |
|  | 9.2 | Demonstrates the proper positioning and attachment of the baby on the breast |  |  |
|  | 9.3 | Gives immunizations according to government policy if possible |  |  |
|  | Achieved: Yes = 1, No = 2 | | |  |
| 10. The provider counsels the mother/parents on baby’s care. (Observe if the provider counsels the mother and father if present, on the following topics) | 10.1 | Importance of early initiation and exclusive breast feeding, |  |  |
|  | 10.2 | positioning and attachment of the baby to the breast |  |  |
|  | 10.3 | Importance of keeping the baby warm |  |  |
|  | 10.4 | Completion of immunization |  |  |
|  | 10.5 | Protecting the baby from infection through hand washing and personal hygiene |  |  |
|  | 10.6 | Care of the umbilical cord |  |  |
|  | 10.7 | Bathing the baby |  |  |
|  | 10.8 | Recommending another visit for the mother and baby for next visit after delivery |  |  |
|  | 10.9 | Pay equal attention for male and female baby. |  |  |
|  | Achieved: Yes = 1, No = 2 | | |  |
| 11.The provider advises the mother/parents on baby’s danger signs.( Observe if the provider with one client) | 11.1 | Advises the mother/parents on the following danger signs for the baby: breathing difficulties and chest in-drawing : |  |  |
|  | 11.2 | (rapid breathing , retractions and grunting), |  |  |
|  | 11.3 | convulsions, |  |  |
|  | 11.4 | fever/ hypothermia, |  |  |
|  | 11.5 | poor suckling or reluctant to eat / feeding, |  |  |
|  | 11.6 | umbilical sepsis ( Redness / swelling/cord discharge) |  |  |
|  | 11.7 | Tells the mother if any of the above signs are present, she needs to bring the baby to the health care facility immediately |  |  |
|  | Achieved: Yes = 1, No = 2 | | |  |

|  | Total Score | Observed score | Achievement score | Proportion |
| --- | --- | --- | --- | --- |
| 1. Standard / Components | 11 |  |  |  |
| 2. Activities | 109 |  |  |  |

1. **Procedure done by**

| **a. Designation of the provider** | **b. which part of the procedure done** |
| --- | --- |
| **1.** | **1.** |
| **2.** | **2.** |
| **3.** | **3.** |
| **4.** | **4.** |
| **5.** | **5.** |
| **6.** | **6.** |

**Code list for designation of the provider:** 01=Consultant/Specialist in Ob/Gyn, 02=MO/Assistant Register, 03=Consultant/Specialist in Anaesthesia, 04=Consultant/Specialist in Paediatrics, 05=SSN/SN, 06=FWV/Senior FWV, 07=HA/SACMO/ MA/ Paramedics, 08= FWA, 09= CHCP/CSBA/ Community volunteer, 10=Assistant Nurse/ Student nurse , 11= ANA/Nurse AID/FMA/ Aya/ Dai nurse/ OT boy, 12= MT, 13=Sweeper/Cleaner/MLSS/Ward boy/Driver,

14= Others (specify_________________________________________________)

1. **Particulars of the primary provider:**

| 1. Sex Male = 1, Female = 2 |  | 4. Years of service | ­­­­Yrs |
| --- | --- | --- | --- |
| 2. Designation |  | 5. Years of service in this facility | Yrs |
| 3. Professional qualification/ Training | a. | b. | c. |

**Code list for Qualification:** 01=FCPS/MCPS/DGO, 02=MBBS, 03=Post graduate training, 04= EOC training, 05=Basic training (FWV/SACMO/Paramedics), 06= Basic training (CHCP/HA), 07=Diploma /BSC in nursing, 08=Midwifery, 09=SBA/TBA/CSBA training, 10=Any other short training, 11=Study in nursing, 12= Others (specify________________________________________________________________________)

1. **Particulars of the Mother:** Collect information from the health care provider at the end of the observation

| 1. Age | Yrs | 2. Para (+Abortus/miscarriage) |  |
| --- | --- | --- | --- |
| 3. Gravida |  | 4. Gestational age | Weeks |
| 5. First pregnancy  Yes = 1 , No = 2 |  | 6. Multiple Pregnancy Yes = 1 , No = 2 |  |
| 7. Type of delivery NVD=1, CS=2, Miscarraige =3, Others ____________________________________________________=4 | | | |
| 8. Any high risk indicator | a. | b. | c. |

**(Gravida**indicates the number of times the mother has been pregnant, regardless of whether these pregnancies were carried to term. A current pregnancy, if any, is included in this count. **Para** indicates the number of >20 wks births (including viable and non-viable i.e. stillbirths). Pregnancies consisting of multiples, such as twins or triplets, count as ONE birth for the purpose of this notation. **Abortus**is the number of pregnancies that were lost for any reason, including induced abortions or miscarriages. The abortus term is sometimes dropped when no pregnancies have been lost. Stillbirths are not included.)

**Code list for High risk factor:** 01=Previous C/S, 02=Pre-eclampsia /Eclampsia, 03=Bad obstetric history, 04= Malpresentation, 05=Sub-fertility, 06=Oligo-hydramnios, 07= Post dated , 08=Incomplete abortion, 09=Fetal distress, 10=Obstructed labor,11= PROM/ Leaking membrane,12= Multiple pregnancy,13=Home trialed, 14=Other Medical problem,15=PV bleeding,16= others (specify_______________________)

| 1. **Comments** |
| --- |
|  |

**Observation End Time: |___||___|:|___||___|**

Signature of the Observer: __________________________ **Date:** ___/___/2014

Signature of the Supervisor: __________________________ **Date:** ___/___/2014

Signature of the Data entry personnel: ________________________ **Date:** ___/___/2014
