# Supplementary material for: Quality of maternal and newborn healthcare services in two public hospitals of Bangladesh: identifying gaps and provisions for improvement
Source: BMC Pregnancy Childbirth. 2019 Dec 10;19:488. doi: 10.1186/s12884-019-2656-1 (PMC6905111; doi:10.1186/s12884-019-2656-1)
Supplement: Supplementary file 8 — Additional file 8. Checklist_Sick Newborn Care.docx (Sick newborn care checklist). [file 12884_2019_2656_MOESM8_ESM.docx]

**Appendix V: Checklists to observe quality of care of MNH Cases attending the Health Facility**

**International Centre for Diarrhoeal Disease Research, Bangladesh (icddr,b)**

**AREA 5: SICK NEWBORN CARE**

**Facility Name: _____________________________________ Facility Type: ______________________________**

**District: _________________________________ Upazilla: _____________________**

**UFI of the facility:** |___|___|___|___|___|___|___|___|

**Place of observation: ____________________________________**

**Code list:** 01= OPD/EPI room, 02=Ward/Cabin, 03=ANC room, 04=Labor/Delivery room, 05=OT,

06=Nurse/ SACMO/CHCP Room, 07= Others (specify_______________________________)

**Assessment Type:** (BASELINE 🞎/PERIODIC🞎)

**Phase of Data collection:** Phase I 🞎/Phase II 🞎/Phase III 🞎

**Name of the Observer** ___________________________________

**Case no:** |___|___| **Patient no:** |___|___|___|___|

**Date:** ___/___/2014  **Observation Start Time: |___||___|:|___||___|**

**Operational definition:**

- **Done**: Performs the step or task according to the standard procedure or guidelines.
- **Not done:** Unable to perform the step or task according to the standard procedure or guidelines.
- **Not applicable**: Step or task not applicable for that particular patient during evaluation by observer.

| **PERFORMANCE STANDARDS** |  | **VERIFICATION CRITERIA** | **Observation**  **[Done=1, Not done=0,**  **Not applicable=9** | **COMMENTS** |
| --- | --- | --- | --- | --- |
| 1. Provider manages sick newborns correctly. (Review clinical records and observe a provider giving care to at least one neonate with each selected condition.) | 1.1 | Demonstrate good communication skill while talking to the mother and family members |  |  |
|  | 1.2 | Washes hands with soap and water and dries them in air/with clean towel or uses an alcohol-based solution |  |  |
|  | 1.3 | Ask about relevant history including birth and feeding history of the neonate during diagnosis |  |  |
|  | 1.4 | Always explain the neonates condition to the family |  |  |
|  | 1.5 | Provide relevant and appropriate messages depending on health condition |  |  |
|  | **Achieved: Yes/No (Circle the answer)** | | |  |
| **For preterm/low birth weight neonate-go to question #2**  **Neonatal sepsis- go to question #3**  **Neonatal jaundice- go to question #4**  **Umbilical infection- go to question #5** | | | | |
| **For preterm/low birth weight neonate** | | | | |
| 2. The provider properly makes the diagnosis of a pre-term/low birth weight neonate. | 2.1 | If no cases are available, provider is able to state clinical features for diagnosing pre-term and low birth weight neonates: |  |  |
|  | 2.2 | Washes hands with soap and water and dries them in air/ clean towel or uses an alcohol-based solution |  |  |
|  | **2.3 Determines whether a baby is Low Birth Weight or states that signs/symptoms which include:** | | |  |
|  | 2.3.a | Low birth weight: Weight less than 2500 g, plus |  |  |
|  | 2.3.b | Fontanels are wide, soft |  |  |
|  | 2.3.c | Soft poorly formed ear cartilage |  |  |
|  | 2.3.d | Thin and transparent skin with visible veins |  |  |
|  | 2.3.e | Breast tissue less than 5 mm |  |  |
|  | 2.3.f | Relatively large abdomen |  |  |
|  | 2.3.g | In female baby, labia major does not cover labia minor |  |  |
|  | 2.3.h | In male baby, the testes may not have descended into the scrotum |  |  |
|  | 2.3.i | Sluggish neonatal reflexes (Sucking, Rooting and Moro) |  |  |
|  | 2.4 | Help the mother with breast feeding when the baby is awake and alert, and with correct positioning and attachment, if necessary |  |  |
|  | 2.5 | If the baby cannot be breastfed, have the mother give expressed breast milk using cup/spoon |  |  |
|  | 2.7 | Skin-to-skin contact starts at birth and continued day and night. There may be brief interruptions such as during the baby’s bath and changing nappies. |  |  |
|  | 2.9 | Provide information to family about how to help the mother to provide continuous KMC |  |  |
|  | **Achieved: Yes/No (Circle the answer)** | | |  |
| **For neonatal sepsis** | | | | |
| 3. Provider properly makes the diagnosis of neonatal sepsis and makes appropriate referral. (Verify whether the provider) | 3.1 | Washes hands with soap and water and dries them in air/ clean towel or uses an alcohol-based solution |  |  |
|  | **3.2 Assesses or can describe the following signs/symptoms:** | | |  |
|  | 3.2.a | Poor feeding |  |  |
|  | 3.2.b | Temperature instability (hypothermia [less than 35.5° C/95.9° F]or fever [temperature more than 37.5°C/99.5° F]) |  |  |
|  | 3.2.c | Fast breathing (more than 60/min)/ Sever chest in-drawing |  |  |
|  | 3.2.d | Convulsion |  |  |
|  | 3.2.e | Moves only when stimulated or no movement at all |  |  |
|  | 3.2.f | Any signs of infection: eyes or umbilicus red or swollen and draining pus or foul smelling |  |  |
|  | 3.3 | After possible diagnosis start appropriate intramuscular Antibiotics according to national guideline: injectable Gentamicin (5 mg/kg/day) AND injectable Amoxicillin (50 mg/kg twice /day) |  |  |
|  | 3.4 | If applicable refers the baby with a proper referral slip to higher health facilities |  |  |
|  | **Achieved: Yes/No (Circle the answer)** | | |  |
| **Fort neonatal jaundice** | | | | |
| 1. The provider properly makes the diagnosis of neonatal jaundice & refers if required.( Determine whether the provider does or can verbalize the following) | 4.1 | Washes hands with soap and water and dries them with a clean towel or uses an alcohol based solution |  |  |
|  | 4.2 | Takes thorough history, including details of date and time of delivery |  |  |
|  | 4.3 | Exams the baby in good day light |  |  |
|  | 4.4 | Assesses the baby for yellow discoloration of skin and mucous membranes |  |  |
|  | 4.5 | Finds out the date of appearance and extent of jaundice |  |  |
|  | 4.6 | The blood group of the mother and the baby’s father |  |  |
|  | 4.7 | Refers the baby with a proper referral slip to higher health facilities |  |  |
|  | 4.8 | Anything applied on the umbilicus |  |  |
|  | 4.9 | Refers the baby with a proper referral slip to higher health facilities |  |  |
|  | **Achieved: Yes/No (Circle the answer)** | | |  |
| **For umbilical infection** | | | | |
| 5. The provider properly makes the diagnosis of umbilical infection. (Determine whether the provider perform or can describe the following) | 5.1 | Washes hands with soap and water and dries them in air/ clean towel or uses an alcohol-based solution |  |  |
|  | 5.2 | Takes thorough history, including whether anything were applied to the baby’s umbilicus |  |  |
|  | **5.3 Looks for :** | | |  |
|  | 5.3.a | Any redness, swelling, draining of pus, or foul smell of umbilicus |  |  |
|  | 5.3.b | Any bleeding from the umbilicus |  |  |
|  | **5.5 Identifies severe or localized umbilical infection:** | | |  |
|  | 5.5.a | Severe: redness and swelling of skin extending more than 1 cm beyond the umbilicus and umbilicus draining pus, or foul-smelling |  |  |
|  | 5.5.b | Localized: redness and swelling of skin extending less than 1 cm beyond the umbilicus |  |  |
|  | 5.6 | Determines any emergency condition that needs immediate attention (serious bacterial infection based on umbilical cord condition) |  |  |
|  | 5.7 | Determines presence of any other local bacterial infection |  |  |
|  | 5.8 | Manages severe infection of umbilicus as follows: |  |  |
|  | 5.9 | If skin pustules/blisters are present, treats for skin infection |  |  |
|  | 5.10 | Provides general care as described for a local infection of the umbilicus |  |  |
|  | 5.11 | Manages local infection of umbilicus as follows: |  |  |
|  | **Achieved: Yes/No (Circle the answer)** | | |  |

**Preterm/Low birth weight neonate:**

|  | Total Score | Observe Score | Achievement Score | Proportion |
| --- | --- | --- | --- | --- |
| 1. Standard / Components | 2 |  |  |  |
| 2. Activities | 14 |  |  |  |

**Neonatal sepsis-**

|  | Total Score | Observe Score | Achievement Score | Proportion |
| --- | --- | --- | --- | --- |
| 1. Standard / Components | 2 |  |  |  |
| 2. Activities | 9 |  |  |  |

**Neonatal jaundice**

|  | Total Score | Observe Score | Achievement Score | Proportion |
| --- | --- | --- | --- | --- |
| 1. Standard / Components | 2 |  |  |  |
| 2. Activities | 14 |  |  |  |

**Umbilical infection**

|  | Total Score | Observe Score | Achievement Score | Proportion |
| --- | --- | --- | --- | --- |
| 1. Standard / Components | 2 |  |  |  |
| 2. Activities | 16 |  |  |  |

1. **Procedure done by**

| **a. Designation of the provider** | **b. which part of the procedure done** |
| --- | --- |
| **1.** | **1.** |
| **2.** | **2.** |
| **3.** | **3.** |
| **4.** | **4.** |
| **5.** | **5.** |
| **6.** | **6.** |

**Code list for designation of the provider:** 01=Consultant/Specialist in Ob/Gyn, 02=MO/Assistant Register, 03=Consultant/Specialist in Anaesthesia, 04=Consultant/Specialist in Paediatrics, 05=SSN/SN, 06=FWV/Senior FWV, 07=HA/SACMO/ MA/ Paramedics, 08= FWA, 09= CHCP/CSBA/ Community volunteer, 10=Assistant Nurse/ Student nurse , 11= ANA/Nurse AID/FMA/ Aya/ Dai nurse/ OT boy, 12= MT, 13=Sweeper/Cleaner/MLSS/Ward boy/Driver,

14= Others (specify_________________________________________________)

1. **Particulars of the primary provider:**

| 1. Sex Male = 1, Female = 2 |  | 4. Years of service | ­­­­Yrs |
| --- | --- | --- | --- |
| 2. Designation |  | 5. Years of service in this facility | Yrs |
| 3. Professional qualification/ Training | a. | b. | c. |

**Code list for Qualification:** 01=FCPS/MCPS/DGO, 02=MBBS, 03=Post graduate training, 04= EOC training, 05=Basic training (FWV/SACMO/Paramedics), 06= Basic training (CHCP/HA), 07=Diploma /BSC in nursing, 08=Midwifery, 09=SBA/TBA/CSBA training, 10=Any other short training, 11=Study in nursing, 12= Others (specify________________________________________________________________________)

1. **Particulars of the Mother:** Collect information from the health care provider at the end of the observation

| 1. Age | Yrs | 2. Para (+Abortus/miscarriage) |  |
| --- | --- | --- | --- |
| 3. Gravida |  | 4. Gestational age | Weeks |
| 5. First pregnancy  Yes = 1 , No = 2 |  | 6. Multiple Pregnancy Yes = 1 , No = 2 |  |
| 7. Type of delivery NVD=1, CS=2, Miscarriage =3, Others ____________________________________________________=4 | | | |
| 8. Any high risk indicator | a. | b. | c. |

**(Gravida**indicates the number of times the mother has been pregnant, regardless of whether these pregnancies were carried to term. A current pregnancy, if any, is included in this count. **Para** indicates the number of >20 wks births (including viable and non-viable i.e. stillbirths). Pregnancies consisting of multiples, such as twins or triplets, count as ONE birth for the purpose of this notation. **Abortus**is the number of pregnancies that were lost for any reason, including induced abortions or miscarriages. The abortus term is sometimes dropped when no pregnancies have been lost. Stillbirths are not included.)

**Code list for High risk factor:** 01=Previous C/S, 02=Pre-eclampsia /Eclampsia, 03=Bad obstetric history, 04= Malpresentation, 05=Sub-fertility, 06=Oligo-hydramnios, 07= Post dated , 08=Incomplete abortion, 09=Fetal distress, 10=Obstructed labor,11= PROM/ Leaking membrane,12= Multiple pregnancy,13=Home trialed, 14=Other Medical problem,15=PV bleeding,16= others (specify_______________________)

| 1. **Comments** |
| --- |
|  |

**Observation End Time: |___||___|:|___||___|**

Signature of the Observer: __________________________ **Date:** ___/___/2014

Signature of the Supervisor: __________________________ **Date:** ___/___/2014

Signature of the Data entry personnel: ________________________ **Date:** ___/___/2014
